# Nuclear PKM2 promotes the progression of oral squamous cell carcinoma by inducing EMT and post-translationally repressing TGIF2

**DOI:** 10.18632/oncotarget.25850

**Published:** 2018-09-18

**Authors:** Fumie Tanaka, Shohei Yoshimoto, Kazuhiko Okamura, Tetsuro Ikebe, Shuichi Hashimoto

**Affiliations:** ^1^ Section of Pathology, Department of Morphological Biology, Division of Biomedical Sciences, Fukuoka Dental College, Tamura, Sawara-ku, Fukuoka 814-0193, Japan; ^2^ Section of Oral Surgery, Department of Oral and Maxillofacial Surgery, Division of Oral and Medical Management, Fukuoka Dental College, Tamura, Sawara-ku, Fukuoka 814-0193, Japan

**Keywords:** PKM2, EMT, TGIF2, oral squamous cell carcinoma, Warburg effect

## Abstract

Pyruvate kinase M2 (PKM2), a glycolytic enzyme, acts as a metabolic function leading to an energy production critical for cancer progression, known as Warburg effect. In this study we showed a pivotal role of PKM2 acting as a non-metabolic function to promote cancer cell progression in human oral squamous cell carcinoma (OSCC) through the induction of epithelial-mesenchymal transition (EMT), which is crucial for the potential in cancer cell invasion, and post-translational TGIF2 degradation. PKM2 immunoreaction was strong in the cytoplasm of invasive cancer cells, and distinct in the nucleus of spindle-shaped cancer cells with EMT characteristics. TGIF2 nuclear immunoreaction was seen in dysplastic epithelial cells but was repressed in cancer cells. *In vitro* analyses, cytoplasmic expression of PKM2 was translocated into the nucleus in human OSCC derived HSC-4 and SAS cells when EMT was stimulated. In addition, nuclear expression of TGIF2 was distinctively repressed in EMT induced HSC-4 and SAS cells. We recognized a mismatch in TGIF2 protein and mRNA expression in EMT induced HSC-4 and SAS cells and found that TGIF2 protein was post-translationally degraded through a ubiquitin proteasome system by an MG132 proteasome inhibition assay. Finally, promotion of HSC-4 and SAS cell progression by PKM2 was recognized in PKM2 knockdown assays. Thus, we clarified a new mechanism of non-metabolic function of PKM2 to promote the progression of OSCC through PKM2 nuclear translocation, subsequently induced EMT, and post-translationally repressed TGIF2 expression by a ubiquitin proteasome system.

## INTRODUCTION

Oral cancers are the sixth most common cancers in the world [1]. Among them, oral squamous cell carcinoma (OSCC) accounts for more than 90% of oral cancers [2]. OSCC sometimes metastasizes to cervical lymph nodes. When metastasis occurs, it shows poor prognosis [3]. In tumor invasion and metastasis, epithelial-mesenchymal transition (EMT) plays an essential role [4, 5]. EMT has been primarily known as a phenotypic change during embryonic development, tissue remodeling and wound healing [6]. When EMT occurs, cells lose intercellular adhesion, alter morphology to spindle-shaped appearance, and increase mobility [6].

Cancer cells metabolize glucose by glycolysis even in the aerobic state although this metabolic process is less efficient for ATP and energy production. This altered metabolism was discovered by Warburg and known as Warburg effect. Warburg effect is considered to be a great advantage for cancer cells to survive and proliferate in the unique hypoxic cancer microenvironments [7, 8].

Pyruvate kinase (PK), an important glycolytic enzyme, transfers phosphate from phosphoenolpyruvate to adenosine diphosphate, and generates pyruvate and ATP [9]. In mammals, PK is encoded by two genes, *PKLR* and *PKM*, and has a total of four isoforms. *PKLR* gene encodes liver-type PK (PKL) and red blood cell PK (PKR) isoforms. PKL is expressed in the liver and kidney, and PKR is expressed in red blood cells [10]. *PKM* gene encodes PKM1 or PKM2. PKM1 is expressed in the differentiated tissue, such as brain and muscle. PKM2 is expressed in the developing or proliferating tissue, including spleen, lung and cancers [9, 10, 11]. In some previous studies it was reported that PKM2 was upregulated in cancers [12, 13]. In cancer cells, PKM2 tetramer acts as a glycolytic enzyme in the cytoplasm. On the other hand, PKM2 dimer can translocate to the nucleus and functions differently as a non-metabolic coactivator [9, 10–13].

TGF-β-induced factor homeobox 2 (TGIF2) is a transcriptional factor and activates *CDH1* expression in epithelial cells. In colorectal cancer, interaction between PKM2 dimer and TGIF2 in the nucleus was reported to contribute to EMT induction [13]. However, the characterization or regulation of PKM2 and TGIF2 in OSCC has not yet been fully elucidated.

In this study we clarified a new mechanism of non-metabolic PKM2 function in promotion of OSCC progression through PKM2 nuclear translocation and subsequently induced EMT and post-translationally regulated TGIF2 expression by a ubiquitin proteasome system.

## RESULTS

### PKM2 and TGIF2 expression in human OSCC

**Figure 1:**
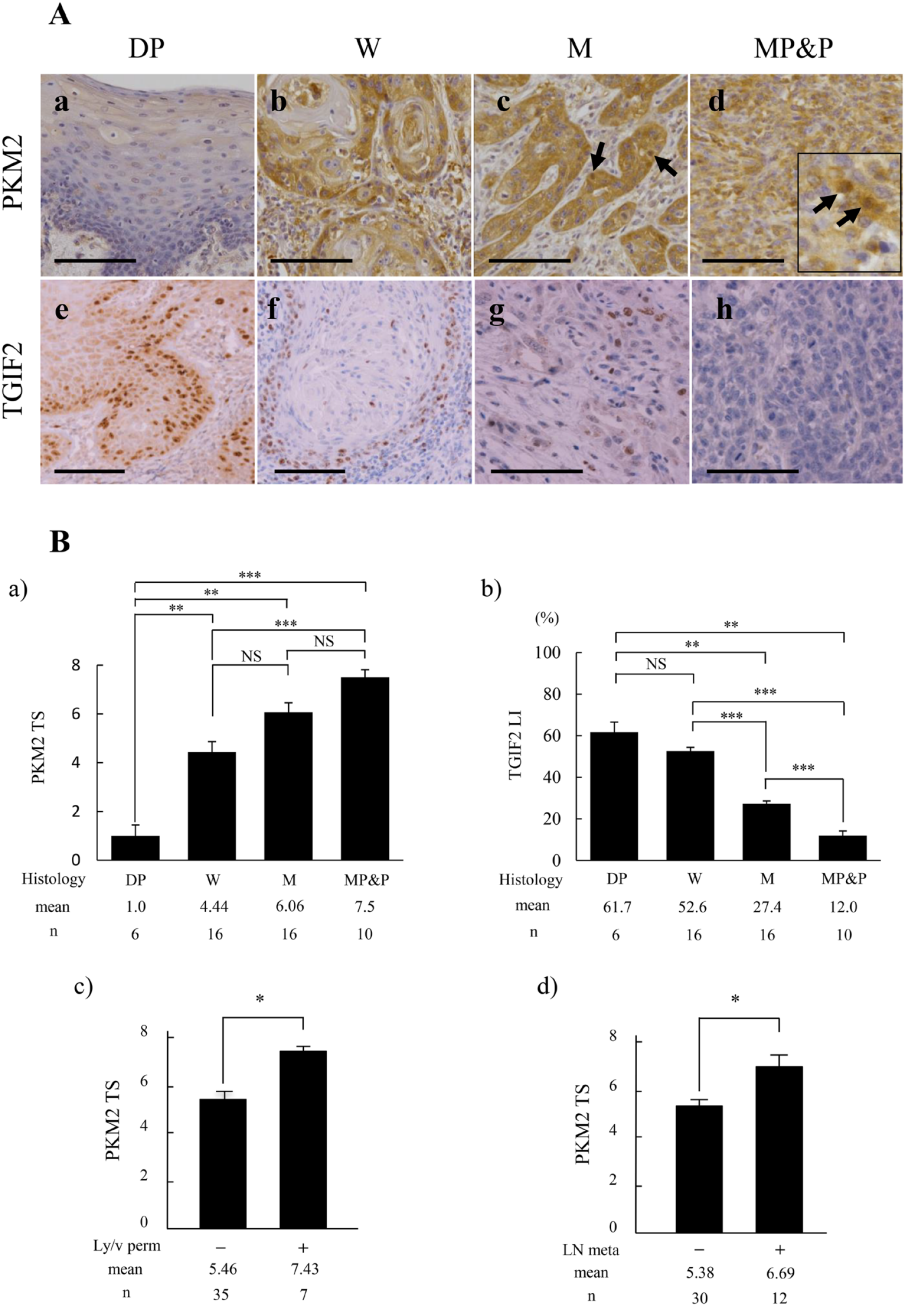
Comparison of PKM2 and TGIF2 expression with clinicopathological indices by immunohistochemical analyses in oral epithelial dysplasia (DP) and each differentiation of oral squamous cell carcinoma (SCC) **(A)** Expression of PKM2 is shown in DP (a), well differentiated SCC (W) (b), moderately differentiated SCC (M) (c), and moderately to poorly and poorly differentiated SCC (MP&P) (d). PKM2 immunoreaction is weak in DP (a), but apparent in W especially in the periphery of each cancer cell nest (b), and strong in the invasive or poorly differentiated cancer cells (c, d). Nuclear expression of PKM2 is shown by arrows in (c) and inset of (d). Expression of TGIF2 is shown in DP (e), W (f), M (g), and MP&P (h). TGIF2 expression is apparent in the nuclei of basal or parabasal cells in DP (e) but is repressed in SCC (f-h). Scale bars: 100 μm. **(B)** Analyses of the relationship between PKM2 TS (a, c, d) or TGIF2 LI (b) and clinicopathological indices. PKM2 TS is significantly higher in SCC than in DP, and gradually increase in accordance with the poor differentiation of SCC (a). A significant difference is seen between W and MP&P (a). TGIF2 LI is significantly decreased in accordance with the poor differentiation of SCC although there is no significant difference between DP and W (b). PKM2 TS is significantly higher in Ly/v permeation (perm) (+) and LN metastasis (meta) (+) than in Ly/v perm (-) and LN meta (-), respectively. (d). Statistical analyses were done by the methods described in materials and methods. Statistical significance was set as ^*^p<0.05, ^**^p<0.01 and ^***^p<0.001.

Immunohistochemically, PKM2 expression was weak or not apparent in dysplasia (DP) (Figure 1A; a), but apparent in OSCC (Figure 1A; b-d). The immunoreaction was present mainly in the cytoplasm of peripheral cells of each cancer cell nest in well (W) differentiated OSCC (Figure 1A; b). The strong immunoreaction was seen in the invasive and poorly differentiated cancer cells in moderately (M), and moderately to poorly and poorly (MP&P) differentiated OSCC (Figure 1A; c and d). Intranuclear expression of PKM2 became to be apparent especially in the poorly differentiated cancer cells with a spindle-shaped appearance, showing EMT characteristics (Figure 1A; arrows in c and d inset). The EMT induction in these spindle-shaped cancer cells was confirmed by the weak or negative staining of E-cadherin and the positive staining of vimentin (Supplementary Figure 1). On the other hand, TGIF2 expression was apparent in the nuclei of basal and parabasal cells in DP (Figure 1A; e), but the expression was decreased in accordance with the poor differentiation of OSCC (Figure 1A; f-h). In addition, these PKM2 and TGIF2 were predominantly expressed in tumor cells, and this was confirmed by the following dual immunofluorescent staining against the same case shown in Figure 1A; g. Namely, TGIF2 was expressed in the tumor cells that expressed basal cell marker p63 (Supplementary Figure 2; a, b, c) and epithelial cell marker cytokeratin (Supplementary Figure 2; d, e, f). Furthermore, TGIF2 was expressed in the tumor cells that expressed PKM2 (Supplementary Figure 2; g, h, i).

The correlation between PKM2 or TGIF2 expression and clinicopathological factors was determined by the mean value of Total Score (TS) or Labeling Index (LI) of each immunoreactivity, respectively, and the difference was statistically analyzed as described in materials and methods. The PKM2 TS in each differentiation of OSCC was significantly higher than that in DP, and the PKM2 TS in OSCC was gradually increased in accordance with the poor differentiation showing a statistical difference in between W and MP&P (Figure 1B; a). In contrary to this, the TGIF2 LI in W of OSCC was lower than that in DP although there was no statistical significance between the two, and the TGIF2 LI in OSCC was significantly decreased in accordance with the poor differentiation (Figure 1B; b). Furthermore, the higher score of PKM2 TS was significantly correlated with the lymphatic and/or vascular (Ly/v) permeation (Figure 1B; c) and lymph-node (LN) metastasis (Figure 1B; d).

### *In vitro* EMT induction in HSC-4 and SAS cells and the changes of PKM2 subcellular localization and TGIF2 nuclear expression

**Figure 2:**
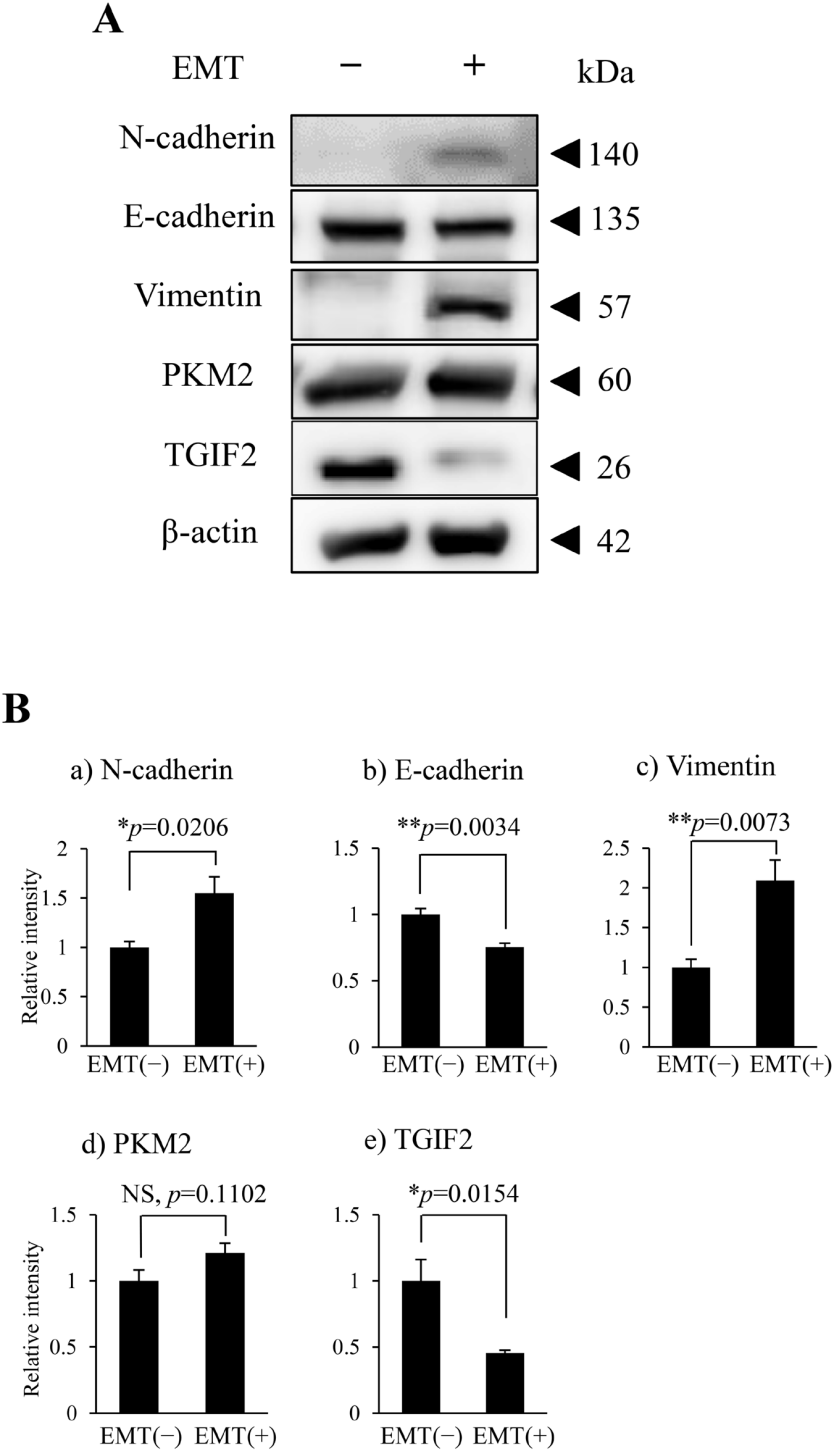
PKM2 and TGIF2 expression in HSC-4 cells in the EMT unstimulated condition (EMT (-)) and stimulated condition (EMT (+)) **(A)** Western blots of N-cadherin, E-cadherin, vimentin, PKM2 and TGIF2 in the EMT(-) and EMT(+). Molecular weight is pointed by an arrowhead. **(B)** Results of densitometry analyses against the bands shown in A. N-cadherin (a) and vimentin (c) are significantly upregulated but E-cadherin (b) is significantly downregulated in EMT (+). TGIF2 is significantly downregulated (e) and PKM2 shows a tendency of increase but no significant difference (d) in EMT (+). Statistical significance assessed by student’s t-tests is indicated by ^*^p<0.05 and ^**^p<0.01.

**Figure 3:**
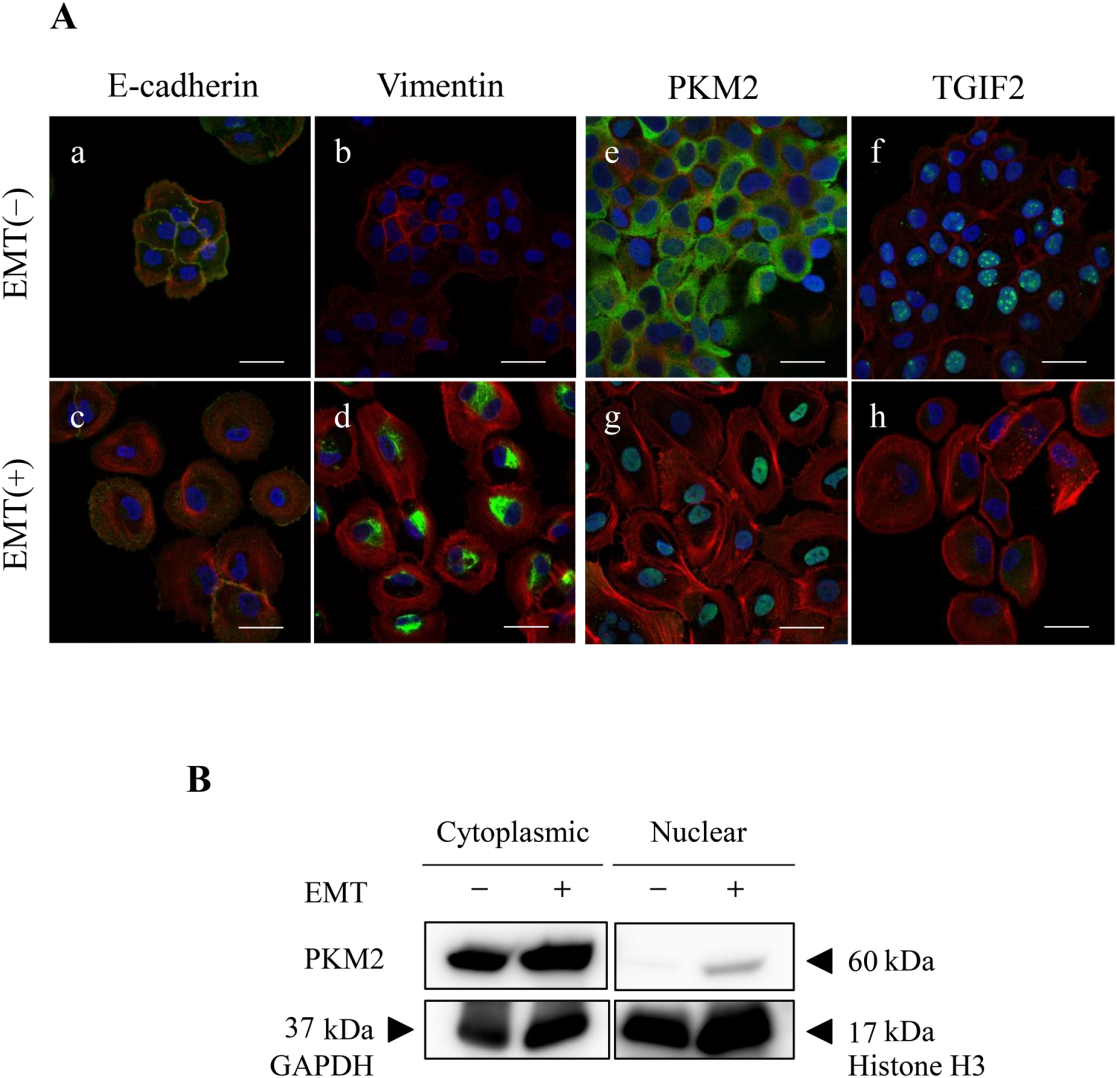
EMT induction alters subcellular localization of PKM2 and expression of TGIF2 **(A)** Immunofluorescent cytochemical staining of E-cadherin (a, c: green), vimentin (b, d: green), PKM2 (e, g: green), TGIF2 (f, h: green) and F-actin (a-h: red), in HSC-4 cells in EMT (-) (upper panels; a, b, e, f) and EMT (+) (lower panels; c, d, g, h). Merged images with nuclear DAPI staining (blue) are shown. EMT induction is confirmed by the decrease of E-cadherin (c) and increase of vimentin (d) expression in EMT (+). Translocation of PKM2 from the cytoplasm (e) to the nucleus (g) and distinct decrease of TGIF2 nuclear expression (h) are recognized in EMT (+). Scale bars: 20 μm. **(B)** PKM2 subcellular expression in HSC-4 cytoplasmic and nuclear fractions in EMT (-) and (+) by western blotting analysis. PKM2 immunoblot is distinct in EMT (+) but not apparent in EMT (-) in the HSC-4 cell nuclear fraction. GAPDH: Cytoplasmic intrinsic marker, Histone H3: Nuclear intrinsic marker. Molecular weight is pointed by an arrowhead.

**Figure 4:**
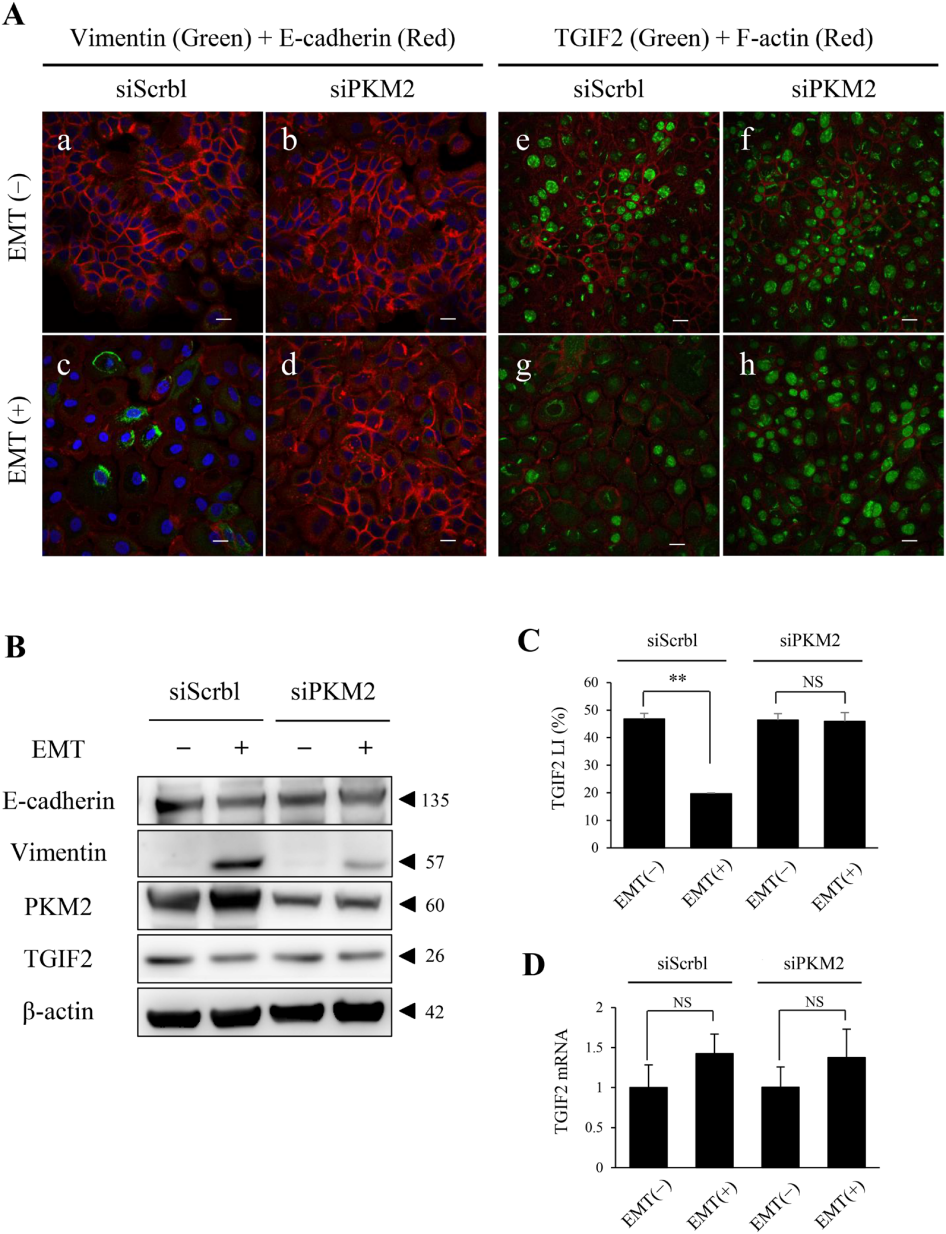
PKM2 knockdown in HSC-4 cells in EMT (+) results in the rescue of TGIF2 expression **(A)** Dual immunofluorescent cytochemical staining of vimentin (green) and E-cadherin (red) , and TGIF2 (green) and F-actin (red) for HSC-4 cells in EMT(-) (upper panels; a, b, e, f) or EMT(+) (lower panels; c, d, g, h) with siScramble (siScrbl) (a, c, e, g) or siPKM2 (b, d, f, h) transfection. EMT induction is clearly inhibited by siPKM2 in EMT (+), which is shown by the repression of vimentin expression in EMT (+) with siPKM2 (d). TGIF2 nuclear expression is distinctively repressed in EMT (+) with siScrbl (g). Surprisingly, this TGIF2 nuclear repression is rescued in EMT (+) with siPKM2 (h). Scale bars: 20 μm. **(B)** Western blotting analyses to support the rescue of TGIF2 repression by PKM2 knockdown in HSC-4 cells in EMT (+) shown in A. The TGIF2 band in EMT (+) with siScrbl is weaker than that in EMT(-) with siScrbl, which supports the loss of TGIF2 expression in the EMT induction. The difference in the TGIF2 bands is not apparent in between EMT (-) and EMT (+) with siPKM2. Molecular weight is pointed by an arrowhead. **(C)** TGIF2 nuclear expression analyses in HSC-4 cells in EMT (-) or (+) with siScrbl or siPKM2 transfection. TGIF2 LI is significantly decreased in EMT (+) with siScrbl compared to that in EMT (-) with siScrbl. However this TGIF2 LI reduction was inhibited in EMT (+) with siPKM2 compared to that in EMT (+) with siScrbl. **(D)** Real-time qPCR analyses show statistically no significant changes in the TGIF2 mRNA expression in HSC-4 cells in between EMT (-) and EMT (+) with siScrbl or siPKM2 (n=3). These results reveal a mismatch in between the TGIF2 protein and mRNA expressions in EMT induced HSC-4 cells, and the TGIF2 nuclear protein expression is maintained by the PKM2 knockdown in EMT induced HSC-4 cells. Student's t-test was used for statistical evaluations. NS: No significance in the statistical analysis.

To support the EMT induction and clarify the role of PKM2 or correlation between PKM2 and TGIF2 expression in human OSCC, especially in the poorly differentiated and spindle-shaped cancer cells, we performed the EMT induction in HSC-4 and SAS cells according to the protocol described in materials and methods. HSC-4 and SAS cells with the EMT stimulation (EMT (+)) changed the morphology from the round- or oval-shaped appearance to the spindle-shaped one (Supplementary Figure 3 for HSC-4 cells, The same data for SAS was not shown), and the EMT induction in HSC-4 and SAS cells with the EMT (+) was confirmed by a significant increase of N-cadherin and/or vimentin and a significant decrease of E-cadherin expression in the immunofluorescent cytochemistry (Figure 3A; a-d, Supplementary Figure 4A; a-d for HSC-4, and Supplementary Figure 5C; a-d for SAS), and western blotting and densitometry analyses (Figure 2A, 2B; a-c for HSC-4, Supplementary Figure 5A for SAS). The EMT induced HSC-4 and SAS cells showed a mild increase of PKM2 expression without statistical difference but a significant decrease of TGIF2 expression in the western blotting, densitometry analyses or LI comparison (Figure 2A, 2B; d, e, Figure 4C (siScrbl) for HSC-4, Supplementary Figure 5A for SAS). However, no statistically significant differences were seen in both PKM2 and TGIF2 mRNA expression in between EMT (-) and (+) conditions by RT-qPCR analyses (Figure 4D (siScrbl) for HSC-4, Supplementary Figure 5B for SAS), suggesting a mismatch in between TGIF2 protein and mRNA expression. Interestingly, the change of subcellular localization of PKM2 expression from the cytoplasm to the nucleus (Figure 3A; e, g, Supplementary Figure 4B; a, b for HSC-4, Supplementary Figure 5C; e, g for SAS) and a distinct repression of nuclear TGIF2 expression (Figure 3A; f, h, Supplementary Figure 4B; c, d for HSC-4, Supplementary Figure 5C; f, h for SAS) were seen in the immunofluorescent cytochemical staining analyses. The repression of nuclear TGIF2 expression in the nucleus showing localized expression of PKM2 in EMT induced HSC-4 cells was also confirmed by the TGIF2 and PKM2 dual immunocytochemistry (Supplementary Figure 6). In addition, distinct but no apparent nuclear PKM2 immunoblot was confirmed in the HSC-4 cell nuclear fraction in EMT (+) and EMT (-) conditions, respectively (Figure 3B). These results suggested that PKM2 translocated to the nucleus from the cytoplasm and simultaneously nuclear TGIF2 expression was distinctively repressed when EMT was inducted in HSC-4 and SAS cells.

### PKM2 is essential for EMT induction

To investigate the role of PKM2 in EMT in OSCC progression, we analyzed the PKM2 function in HSC-4 and SAS cells with or without EMT induction by a PKM2 inhibition assay using a small interfering RNA (siRNA). We preliminary confirmed that PKM2 expression in HSC-4 cells was significantly inhibited by siRNA for PKM2 (siPKM2) in both EMT uninduced and induced conditions (Supplementary Figure 7A, 7B). PKM2 depletion resulted in the inhibition of EMT induction in HSC-4 cells by repressing vimentin and maintaining E-cadherin expression (Figure 4A; a-d, Supplementary Figure 8A; a-d). The same results were also obtained in the SAS cell immunocytochemical analyses (data not shown). Interestingly, the repression of TGIF2 nuclear expression in EMT (+) with siScrbl transfection (Figure 4A; g, Supplementary Figure 8B; b) was rescued when PKM2 was knocked down in EMT (+) with siPKM2 transfection (Figure 4A; h, Supplementary Figure 8B; d) in HSC-4 cells. The same result of TGIF2 rescue was also seen in SAS cells by immunocytochemical analyses (data not shown). Western blotting analyses and LI comparison also showed the same results that the repression of TGIF2 expression in EMT induced HSC-4 cells with siScrbl transfection was rescued when the EMT induction was inhibited by siPKM2 transfection (Figure 4B, 4C). However, RT-qPCR analyses showed that no significant differences in the expression levels of TGIF2 mRNA were seen in HSC-4 cells in between EMT (-) and EMT (+) in both PKM2 non-depleted (siScrbl transfected) and depleted (siPKM2 transfected) conditions (Figure 4D). The same result of no significant difference of TGIF2 mRNA expression in between EMT(-) and EMT (+) in both PKM2 non-depleted and depleted conditions was also seen in SAS cells (data not shown). These results revealed a mismatch in between TGIF2 protein and mRNA expressions in EMT induced HSC-4 and SAS cells and suggested that TGIF2 protein expression was regulated and degraded post-translationally by a direct or indirect effect of PKM2 in EMT induced HSC-4 and SAS cells.

### The TGIF2 expression in EMT induced HSC-4 cells was post-translationally regulated through the proteasome activity

**Figure 5:**
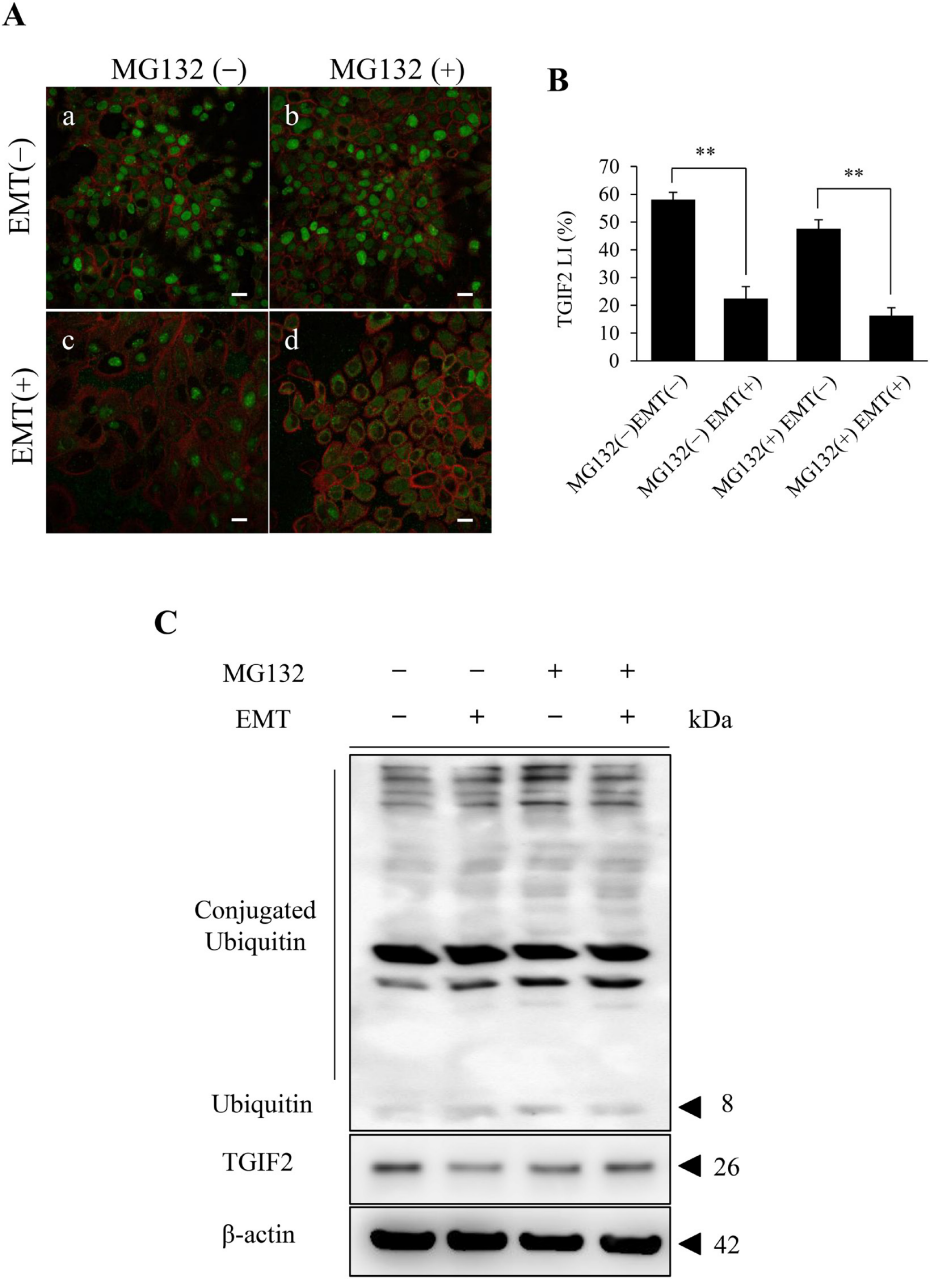
Analyses of the effect of the proteasome inhibitor MG132 on the TGIF2 expression in HSC-4 cells in EMT (+) **(A)** Immunofluorescent cytochemical staining of TGIF2 (green) and F-actin (red) for HSC-4 cells in each culture condition is shown. TGIF2 is largely expressed in HSC-4 cells in their nuclei in EMT (-) in both MG132 untreated (MG132 (-)) (a) and treated (MG132 (+)) (b) conditions. The nuclear TGIF2 expression is distinctively repressed in HSC-4 cells in EMT (+) and MG132 (-) (c). However, the repression of TGIF2 is rescued in HSC-4 cells by the treatment of MG132 in EMT (+) and the immunoreaction is seen largely in the cytoplasm and partly in the nuclei (d). Scale bar: 20 μm. **(B)** Comparison of TGI2 nuclear expression in between EMT (-) and (+) with or without MG132 by TGIF2 LI analyses. TGIF2 LI in EMT (+) and MG132 (-) or (+) is significantly lower than that in EMT (-) and MG132 (-) or (+). These results revealed that TGIF2 nuclear expression is repressed in EMT (+) regardless of MG132 treatment. **(C)** Western blot analyses of TGIF2 in HSC-4 cells in EMT (-) or (+) and MG132 (-) or (+) (n=3). The band of TGIF2 in EMT(+)MG132(-) is weaker than that in EMT(-)MG132(-). However, there is no clear difference in the bands in between EMT(-) MG132(+) and EMT(+)MG132(+). These results reveal MG132 inhibit the repression of TGIF2 expression in EMT (+). Molecular weight is pointed by an arrowhead.

To clarify the mechanisms of TGIF2 reduction in EMT, a post-translational protein regulation, namely, degradation of TGIF2 through the ubiquitin proteasome system was evaluated. The proteasome activity was inhibited by the treatment of MG132, a well-known proteasome inhibitor, in HSC-4 and SAS cells in each EMT (-) or EMT (+) culture condition as described in materials and methods. In the immunofluorescent cytochemical staining analyses, TGIF2 was distinctively seen in the nucleus of HSC-4 cells in both MG132 untreated (MG132 (-)) and treated (MG132 (+)) conditions in the EMT (-) condition (Figure 5A; a, b, Supplementary Figure 9; a, c for HSC-4, Supplementary Figure 10A; a, c for SAS). However, the TGIF2 nuclear expression in HSC-4 cells in the EMT (-) and MG132 (-) condition was remarkably repressed in HSC-4 cells in the EMT (+) and MG132 (-) condition (Figure 5A; a, c, Supplementary Figure 9; a, b for HSC-4, Supplementary Figure 10A; a, b for SAS). LI analyses for TGIF2 nuclear expression also showed significant LI decrease in between MG132(-)EMT(-) and MG132(-)EMT(+) and also in between MG132(+)EMT(-) and MG132(+)EMT(+) (Figure 5B for HSC-4, Supplementary Figure 10B for SAS). Strikingly, this repression of TGIF2 expression in MG132(-)EMT(+) was rescued and the TGIF2 expression was seen mainly in the cytoplasm and focally in the nucleus in HSC-4 and SAS cells in MG132(+)EMT(+) (Figure 5A; c, d, Supplementary Figure 9; b, d for HSC-4, Supplementary Figure 10A; b, d for SAS). In western blotting analyses, the band of TGIF2 in MG132(-)EMT(+) was weaker than that in MG132(-)EMT(-). On the other hand, there were no significant changes in the TGIF2 bands in HSC-4 cells in between MG132(+)EMT (-) and MG132(+)EMT(+) (Figure 5C). Ubiquitin- and conjugated ubiquitin-bind bands were also observed, but those bands in MG132 (+) were more distinct than those in MG132 (-), which revealed the inhibitory effect of MG132 against proteasome activity (Figure 5C). These results suggested that TGIF2 was post-translationally degraded through proteasome activity probably in the cytoplasm in the EMT induced HSC-4 and SAS cells.

### PKM2 played pivotal roles in the OSCC progression

**Figure 6:**
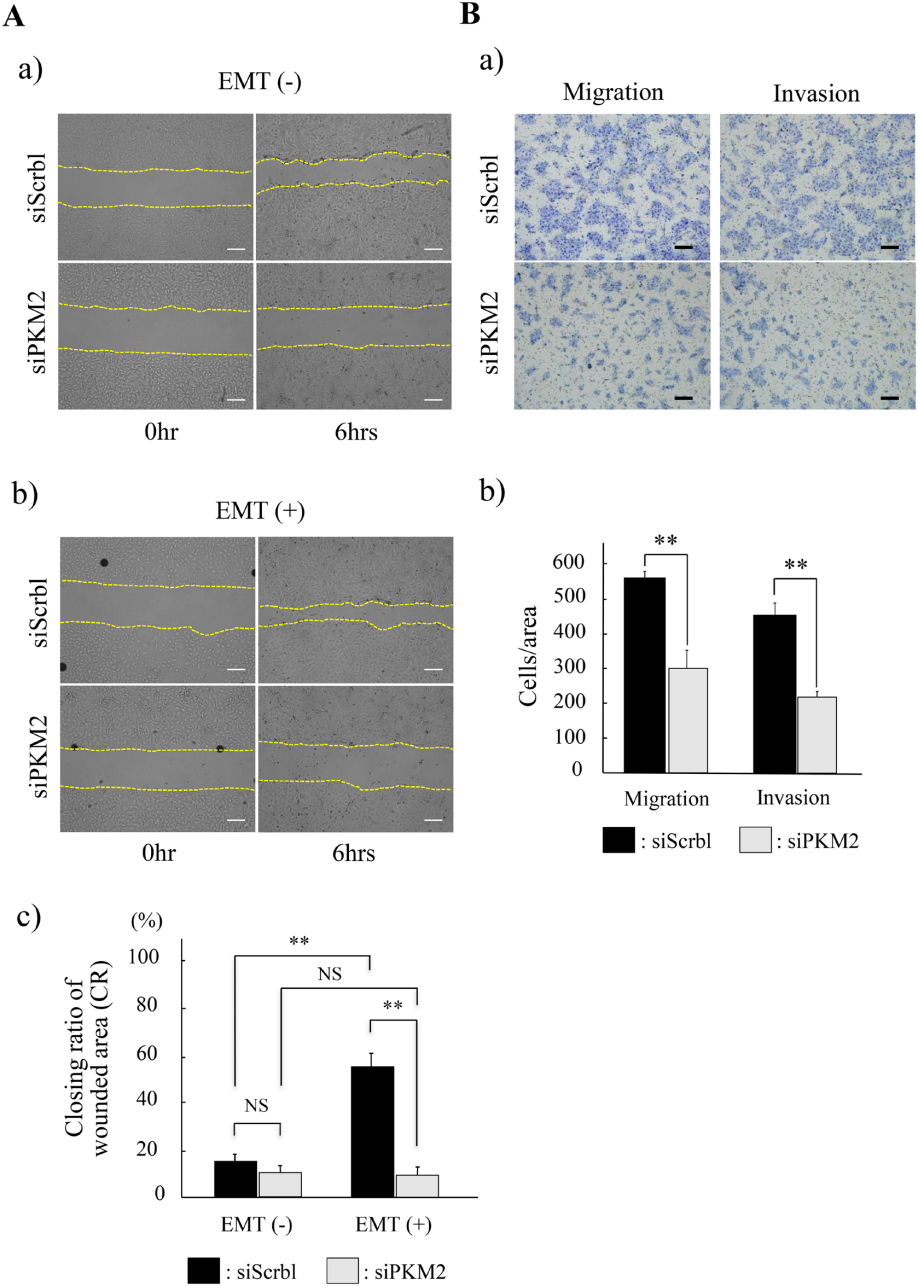
Functional analyses of PKM2 in HSC-4 cells **(A)** Wound healing assay to evaluate the ability of HSC-4 cells migration in siScramble (siScrbl) or siPKM2 transfected cells. The closing pattern of wounded area was displayed in the panels of each EMT unstimulated (EMT (-)) (a) or stimulated (EMT (+)) (b) condition. Upper panels show siScrbl transfected cells and lower panels show siPKM2 transfected cells in EMT (-) (a) or EMT (+) (b). The culture is stopped after 6 hrs (each right column) from the start 0 hr (each left column) (a, b). Bars indicate the closing ratio of wounded area (CR) in siScrbl (black bars) or siPKM2 (gray bars) transfected cells in EMT (-) or EMT (+) (n=6) (c). **(B)** Transwell cell migration and invasion assays for HSC-4 cells in EMT (+). Upper panels show siScrbl transfected cells and lower panels show siPKM2 transfected cells (a). Bars indicate numbers of migrated or invaded cells with siScrbl (black bars) or siPKM2 (gray bars) transfection (b) (n=6). Statistical significance was set as ^*^p<0.05 and ^**^p<0.01 (A, B).

Based on the result that the strong PKM2 immunoreaction was seen in the invasive and poorly differentiated cancer cells in OSCC (Figure 1A; c, d), we hypothesized that PKM2 might be related to the cancer cell migration or invasion. To clarify this PKM2 function, we finally investigated the migration and invasion ability of HSC-4 and SAS cells in the PKM2 uninhibited or inhibited condition. In the wound healing assays, cancer cell migration ability, namely, the closing ratio of wounded area (CR), was increased with (HSC-4) or without (SAS) statistical significance in the EMT stimulated (EMT (+)) condition than in the EMT unstimulated (EMT (-)) condition with siScrbl transfection (Figure 6A; upper panels in a and b, black bars in c for HSC-4, Supplementary Figure 11; upper panels in A and B, black bars in C for SAS). However, no statistical difference in the CR was seen in between the siScrbl and siPKM2 transfection in the EMT (-) condition (Figure 6A; a, EMT (-) in c for HSC-4, Supplementary Figure 11; A, EMT (-) in C for SAS), and no significant increase in the CR was seen in between the EMT (-) and EMT (+) condition with siPKM2 transfection (Figure 6A; lower panels in a and b, gray bars in c for HSC-4, Supplementary Figure 11; lower panels in A and B, gray bars in C for SAS). Strikingly, the CR in the EMT (+) condition with the siScrbl transfection was significantly repressed in the EMT (+) condition with siPKM2 transfection (Figure 6A; b, EMT (+) in c for HSC-4, Supplementary Figure 11C; EMT(+)). These results showed that the increased migration activity in the EMT induced HSC-4 and SAS cells was significantly inhibited by the PKM2 knockdown. The transwell cell migration and invasion assays also showed that PKM2 knockdown significantly repressed the mobility of HSC-4 cells (Figure 6B; a, b). SAS cells revealed same tendency as repression of migration and invasion by PKM2 knockdown but there was no statistical significance (data not shown). These results obtained from our invasion and migration assays revealed that PKM2 promoted the invasion and migration activity of HSC-4 and SAS cells in the EMT state. Finally, we, in this study, clarified a new mechanism of non-metabolic function of PKM2 to promote the progression of OSCC through PKM2 nuclear translocation and subsequently induced EMT and post-translationally repressed TGIF2 expression by a ubiquitin proteasome system.

## DISCUSSION

In this report we highlighted a pivotal non-metabolic function of PKM2 other than its metabolic function of energy production, known as Warburg effect, in the OSCC progression. We observed that the PKM2 expression was significantly increased in accordance with the poor differentiation of OSCC and was strong in the invasive and poorly differentiated cancer cells. In addition, nuclear expression of PKM2 was distinct especially in the spindle-shaped cancer cells showing the EMT characteristics. We also showed that high scores of PKM2 TS were significantly correlated with the lymphatic and/or vascular permeation and lymph node metastasis. From these clinicopathological analyses, we hypothesized that PKM2 might be related to EMT and play some crucial roles in the OSCC progression, and if so, PKM2 might be a useful clinical therapeutic target in addition to a potential biomarker representative of cancer cell activity or metastasis in OSCC.

According to literatures, PKM2 exists and constitutes a tetramer structure in the cytoplasm of cancer cells, and the form is glycolytically active (acts as pyruvate kinase) and important for the cancer metabolism and tumor growth [14]. On the other hand, PKM2 is converted to a dimer inactive form during cancer progression. The dimer of PKM2 regulates gene transcription by acting as a protein kinase in the cancer cell nucleus, and correlates with cancer cell proliferation [15]. Anastasiou et.al. suggested that small-molecule PKM2 activators could promote tetramer formation of PKM2 and suppressed tumorigenesis [16]. In cancer cells, PKM2 translocates to the nucleus from the cytoplasm in response to the epidermal growth factor receptor (EGFR) signaling [17, 18], and is associated with a higher incidence of distant metastases [19, 20]. These reports suggest that PKM2 may play a role in EGFR related metastases. PKM2 is directly bound with EGFR-activated extracellular signal-regulated kinase 2 (ERK2) at PKM2-specific exon 10-encoded Ile 429/Leu 431 through ERK2 docking groove, and is phosphorylated at Ser 37. Phosphorylated PKM2 Ser 37 recruits protein interacting with NIMA (never in mitosis A) -1 (PIN1). PIN1 isomerizes specific phosphorylated Ser/Thr-Pro motifs, that catalyze the conformational switch from *cis* to *trans*, in many substrate proteins including PKM2. This Pin1-mediated *cis*-*trans* isomerization of PKM2 promotes PKM2 binding to importin α5 and translocating to the nucleus. For this nuclear translocation of PKM2, nuclear localization signal (NLS) sequences of PKM2 carboxy-domain encoded by PKM2-specific exon 10, especially NLS-containing Arg 399/400, is essential. Nuclear PKM2 acts as a coactivator of β-catenin to induce c-Myc expression, resulting in upregulation of GLUT1, LDHA and polypyrimidine-tract binding protein (PTB)-dependent PKM2 expression in a positive feedback loop [18–21]. Binding of K433 of nuclear PKM2 to c-Src-phosphorylated Y333 of β-catenin is required for their recruitment to the cyclin D1 gene (*CCND1*) promoter, leading to HDAC3 removal from the promoter, histone H3 acetylation and cyclin D1 expression [17]. EGFR and Pin1 overexpression were also observed in OSCC and were associated with a poor prognosis [22, 23, 24]. Furthermore, Giannoni et. al. suggested that cancer associated fibroblasts (CAFs) promote PKM2 nuclear translocation through both oxidation and Src-mediated phosphorylation, and also demonstrated that nuclear PKM2 was associated with HIF-1α and caused EMT [25]. From these previous reports, Pin1 may also play a role for PKM2 translocation to the nucleus in OSCC, and cell cycle progression by cyclin D1 expression through nuclear PKM2 and β-catenin binding may also be one of the mechanisms of cancer cell progression in OSCC progression. Thus, PKM2 plays a pivotal role in cancer progression in EMT other than the metabolic function called Warburg effect in the anaerobic tumor microenvironments.

On the other hand, relationship of PKM2 and cancer progression remains to be controversial. Some recent studies suggested that PKM2 was not necessary for tumorigenesis and tumor progression [26, 27]. In our PKM2 knockdown assays in HSC-4 and SAS cells, both cancer cell migration and invasion were significantly inhibited. In addition, EMT induction in HSC-4 and SAS cells was also inhibited by PKM2 knockdown. According to these findings in our experiments, cancer cells being in the state of EMT induced by nuclear translocation of PKM2, which is specifically activated by EGFR signaling, was essential for cancer cell progression. Thus, PKM2 may not contribute to such a breast tumor tumorigenesis and progression induced by Brca [26] other than the EGFR signaling pathway.

TG-interacting factor (TGIF) represses transcription by binding directly to DNA or interacts with transforming growth factor β (TGF-β)-activated Smads, leading to repression of TGF-β-responsive gene expression. TGF-β-induced factor homeobox 2 (TGIF2) contains two regions of high sequence identity with TGIF. TGIF2 and TGIF have very similar DNA-binding homeodomains. TGIF2 represses transcription by binding to DNA via a TGIF binding site. TGIF2 also interacts with TGF-β-activated Smads and represses TGF-β-responsive transcription [28]. In colon cancer cells, TGIF2 directly interacts with PKM2 in the nucleus by EMT stimulation, and recruits histone deacetylase 3 to the *CDH1* (E-cadherin) promoter sequence, which subsequently deacetylates histone H3 and suppresses *CDH1* transcription [13]. In our results, TGIF2 expression in the nucleus was apparently decreased in accordance with the poor differentiation of OSCC and was also distinctively decreased in HSC-4 and SAS cells when EMT was induced *in vitro*. This reduction of TGIF2 in HSC-4 and SAS cells by EMT stimulation was rescued by a proteasome inhibitor MG132. Together with our result that there was a divergence between TGIF2 protein and mRNA expression in EMT induced HSC-4 and SAS cells, we concluded that TGIF2 was post-translationally degraded by the proteasome activity in the ubiquitin proteasome system in the state of EMT induced by PKM2. Recently tristetrapolin (TTP), an AU-rich, element-binding protein that regulates mRNA stability, was identified as a new binding partner of PKM2. PKM2 suppresses TTP protein levels by promoting its phosphorylation, ubiquitination, an proteasome degradation, leading to regulation of cell proliferation in breast cancer [29]. This is a supportive evidence for a role of PKM2 in TGIF2 regulation through a ubiquitin proteasome system in OSCC cells. There are also some reports describing other mechanisms of TGIF2 regulation by binding with some microRNAs such as miR-148a [30] and miR-34a [31], which are contributing to skin and gastric cancer progression, respectively. In these reports, TGIF2 expression was lower in cancer cells than in normal cells, and these results are also consistent with our results. These reports suggest there are some different ways of TGIF2 regulation.

Contrary to these reports, in which TGIF2 functioned as an inhibitory manner and loss of TGIF leaded to progression in cancer development, there were a few reports describing the contradictory effect of TGIF2 in cancer development [32, 33]. Imoto et al., reported that TGIF2 gene was overexpressed in ovarian cancer cell lines and might play an important role in the development/or progression of some ovarian tumors through a mechanism of gene amplification [34]. However, they did not mention the TGIF2 protein expression and its relation to the ovarian tumor progression clearly. They also did not show such a post translational regulation of TGIF2 like that we clarified in this report. Moreover, TGF-β signaling plays dual roles in tumor microenvironment whether suppresses or promotes tumor formation [35, 36, 37]. Thus, TGIF2 is affected by TGF-β signaling, and might also act both positive and negative manner. Further investigations are needed on some more types of cancers to reveal the TGIF2 promotive or inhibitory effect on cancer cells.

**Figure 7:**
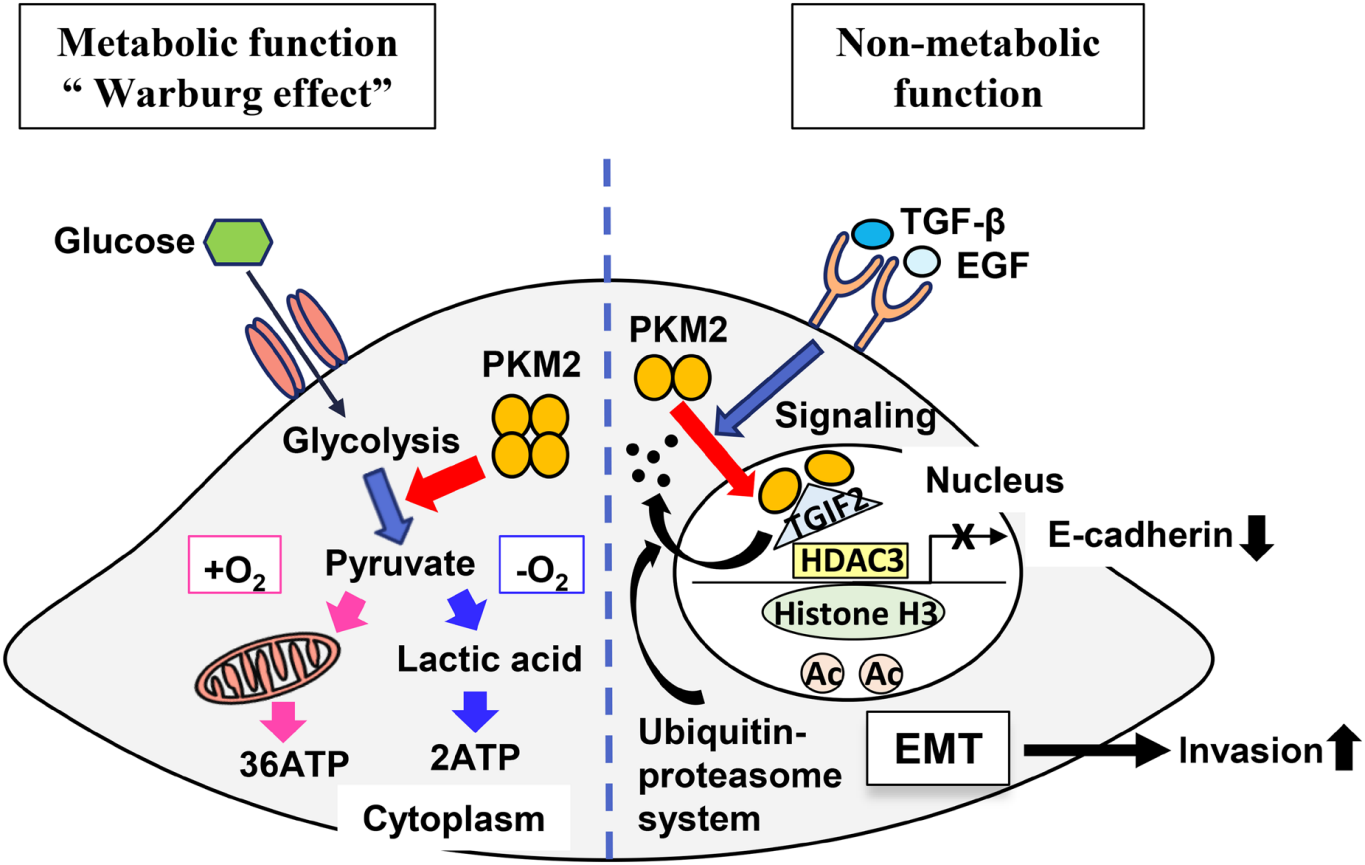
A schematic illustrating the theoretical model revealing roles of PKM2 and TGIF2 regulation Left side is the schematic illustrating the role of PKM2 as a metabolic function. Right side is the schematic illustrating the role of PKM2 as a non-metabolic function. TGIF2 is bound with the dimer of PKM2 in the nucleus and degraded by proteasome activity through a ubiquitin-proteasome system. This facilitates and promotes the recruitment of histone deacetylase 3 (HDAC3) to the *CDH1* (E-cadherin) promoter sequence, which subsequently deacetylates histone H3 and suppresses *CDH1* transcription. Thus, non-metabolic function of PKM2 induces EMT and promote cancer cell invasion.

In summary, we here clarified a pivotal role of PKM2 acting as a non-metabolic function to promote the cancer cell progression in OSCC by translocating to the nucleus and subsequently inducing EMT and repressing TGIF2, and a new mechanism of TGIF2 regulation by PKM2 through a ubiquitin proteasome system in OSCC progression (Figure 7). Thus, PKM2 and its binding partner TGIF2 might be a useful clinical therapeutic target in OSCC in addition to a potential biomarker representative of the cancer cell activity or metastasis in OSCC.

## MATERIALS AND METHODS

### Patients and clinicopathological profiles

This clinical study using the patients’ information was done under the permission of the ethics committee in Fukuoka Dental College. The 48 cases (male/female: 29/19, mean age: 62.1 (range: 25-87)) examined in this study consisted of 6 oral dysplasia (classified by atypical grades as moderate: 3, and severe/carcinoma *in situ*: 3) and 42 oral squamous cell carcinoma (OSCC) (classified by differentiation as well: 16, moderate: 16, moderate to poor and poor: 10). These Japanese patients underwent surgery at Fukuoka Dental College Hospital, Fukuoka, Japan, between 2010 and 2017. The patients were not prescribed chemotherapy or irradiation before surgery. The histological classification was performed according to the criteria of the “WHO Classification of Head and Neck Tumours” (2017) [2]. Histological classification in atypia and differentiation, lymphatic and/or vascular permeation (Ly/v perm) and lymph-node metastasis (LN meta) were adopted as the clinicopathological indices. The clinicopathological profiles of the patients are summarized in Table 1.

**Table 1:** Summary of the clinicopathological characteristics of oral epithelial dysplasia and oral squamous cell carcinoma patients examined

Characteristics	Number of patients
Age	Mean: 62.1 (range: 25-87)
Sex male	29
female	19
Pathological diagnosis	
Oral epithelial Dysplasia (DP)	
Histological grade	
Moderate	3
Severe/Carcinoma *in situ*	3
Oral squamous cell carcinoma (SCC)	
Histological differentiation	
well (W)	16
moderate (M)	16
moderate to poor and poor (MP&P)	10
pT T1	13
T2	18
T3	3
T4	8
pN N0	30
N1	4
N2b	7
N2c	1
pM M0	41
M1	1
pStage I	13
II	14
III	3
IV	12
Ly / v -	35
	7

The 48 cases (male/female: 29/19, mean age: 62.1 (range: 25-87)) examined in this study consist of 6 oral dysplasia (classified by atypical grades as moderate: 3, and severe/carcinoma *in situ*: 3) and 42 oral squamous cell carcinoma (OSCC) (classified by differentiation as well: 16, moderate: 16, moderate to poor and poor: 10). Patient number of each classification of pTNM and pStage is also shown. Lymphatic and/or vascular (Ly/v) permeation was recognized in 7 of 42 patients.

### Antibodies

For the immunohisto- and cyto-chemistry and wester blotting analyses, the following antibodies were used. The primary antibodies; rabbit anti-human PKM2 monoclonal antibody (#4053), rabbit anti-human E-cadherin monoclonal antibody (#3195), rabbit anti-human N-cadherin polyclonal antibody (#4061), rabbit anti-human ubiquitin polyclonal antibody (#3933), rabbit anti-human histone H3 polyclonal antibody (#9715), rabbit anti-human GAPDH monoclonal antibody (#2118) and mouse anti-human β-actin monoclonal antibody (#3700) were purchased from Cell Signaling Technology Inc. (Danvers, MA, USA). Rabbit anti-human TGIF2 monoclonal antibody (ab155948) and mouse anti-bovine vimentin monoclonal antibody (ab8978) were purchased from abcam (Cambridge, UK). The secondary antibodies; Horseradish peroxidase (HRP)-conjugated polymer anti-rabbit and anti-mouse antibodies were purchased from DAKO-Agilent Technologies Co. (Santa Clara, CA, USA). HRP-linked anti-rabbit and -mouse antibodies were purchased from Cell Signaling Technology Inc. (Danvers, MA, USA). Alexa Fluor 594-conjugated goat anti-rabbit IgG and Alexa Fluor 488-conjugated goat anti-mouse IgG antibodies were purchased from Invitrogen-Thermo Fisher Scientific (Waltham, MA, USA).

### Immunostaining for tissues and cells

10% buffered formalin-fixed and paraffin-embedded tissue blocks were cut into 4 μm-thick sections for H.E. and immunohistochemical staining. Antigen retrieval was performed in all sections by treatment with an autoclave at 121°C for 5 min in 0.01M citrate buffer, pH 6.0. Immunostaining was performed by using EnVision/horseradish peroxidase (HRP) kit (DAKO-Agilent Technologies Co., Santa Clara, CA, USA). Briefly, the sections were treated with a 0.1% hydrogen peroxide–methanol solution to inhibit endogenous peroxidase activity and a 5% BSA/TBS to block any non-specific binding of primary antibodies. Subsequently, each section was incubated with the primary antibody against PKM2 (1:800 dilution), TGIF2 (1:200 dilution), E-cadherin (1:200 dilution) or vimentin (1:200 dilution) at 4°C overnight. These sections were then incubated with HRP-conjugated polymer anti-rabbit or anti-mouse antibody. The peroxidase activity was visualized using 0.1% 3, 3’-diaminobenzidine and 0.01% hydrogen peroxide in TBS. For the immunofluorescent staining, after incubation with each primary antibody, the section was incubated with Alexa Fluor 594-conjugated goat anti-rabbit IgG (1:1,000 dilution) or Alexa Fluor 488-conjugated goat anti-mouse IgG (1:1,000 dilution) secondary antibody, followed by nuclear counterstaining with DAPI (1:3000 dilution). Then, sections were mounted using VECTASHIELD (Vector Lab., Burlingame, CA, USA). Photomicrographs were visualized and captured at the appropriate wavelength using a fluorescence microscope (LSM 710, Carl Zeiss Inc.). The images were processed in ZEN 2010B Sp1 Ver. 6.0.0.485 software (Carl Zeiss Inc.). For immunocytochemistry, the same immunostaining procedure described above was applied for cells after fixation with 4% paraformaldehyde (PFA).

### Immunohistochemical assessment

The degree of positivity of immunoreaction in each lesion was determined according to the modified method of the one originally described by Allred et al [38]. Briefly, we randomly chose three areas at the lesion of dysplasia or OSCC section and counted the number of immunoreactive atypical cells for PKM2 in their cytoplasm or nuclei, and for TGIF2 in their nuclei based on at least 300 atypical cells. The percentage of immunoreactive atypical cells was described as proportion score (PS) [scored on a scale of 0-3; 0: 0%, 1: less than 10%, 2: less than 30%, 3: equal to or more than 30%]. Staining intensity was also described as intensity score (IS) (scored on a scale of 0-3; 0: negative, 1: weak positive, 2: intermediate positive, 3: strong positive). The proportion and intensity scores were summed to produce total score (TS = PS + IS) [scored on a scale of 0, 2-6]. The percent rate of positive nuclear expression of TGIF2 (IS; 2, 3) was represented as Labeling Index (TGIF2 LI). Then, the mean score of TS or LI was statistically compared for analyzing the correlation between PKM2 or TGIF2 expression and clinicopathological indices.

### Cell culture

Two human squamous cell carcinoma cell lines established from tongue squamous cell carcinoma patients, HSC-4 (sex: male, age: 64) and SAS (sex and age: non-disclosure), were purchased from JCRB cell bank (Osaka, Japan). Cells were grown in Modified Eagle’s Medium (MEM, Thermo Fisher Scientific, Waltham, MA, USA), supplemented with 10% fetal bovine serum (FBS, PAA Laboratories, Pasching, Austria) at 37°C in 5% CO_2_ / 95% air. Cells were reseeded for the next passage after trypsin (Thermo Fisher Scientific, Waltham, MA, USA) dispersion when they reached ∼ 80% confluency.

### EMT induction and proteasome inhibition assay

EMT was induced in HSC-4 and SAS cells according to the previously reported procedure [13]. Briefly, cells were seeded at a concentration of 5.0 x 10^4^ cells/mL and incubated in a humidified atmosphere at 37°C in 5% CO2/95% air in standard medium for 48 hours. Subsequently, cells were incubated with FBS-free MEM supplemented with 5.0 ng/mL TGF-β1 and 10 ng/mL EGF (Sigma-Aldrich, St. Loius, MO, USA), 100x insulin-transferring selenium (ITS; Thermo Fisher Scientific, Waltham, MA, USA), and 50 nmol/L hydrocortisone (HO533-1G, Tokyo kasei, Tokyo, Japan) for 72 hours. In proteasome inhibition assay in the EMT induction, HSC-4 and SAS cells were cultured in standard medium with 10 μM MG132 (Sigma-Aldrich) for 1 hour. Subsequently, cells were incubated according to the EMT induction protocol with 10 μM MG132 for 3 hours. Then, the EMT induction culture was continued without MG132 and finalized at the time of 72 hours after the EMT induction was started.

### Western blotting analysis

Western blotting analysis was performed as previously described. Briefly, HSC-4 cells were homogenized in an ice-cold lysis buffer and centrifuged at 50,000 x g for 30 min at 4°C. The supernatants (20 μg) were separated on a 4-12% Bis-Tris Plus gel (Thermo Fisher Scientific, Waltham, MA, USA) and transferred to a polyvinyldifluoride membrane (Millipore, Darmstadt, Germany). Immunoblot analyses were performed using rabbit anti-PKM2, rabbit anti-N-cadherin antibodies, rabbit anti-human histone H3 polyclonal antibody, rabbit anti-human GAPDH monoclonal antibody and rabbit anti-human ubiquitin polyclonal antibody (1:1000), and mouse anti-vimentin antibody (1:500). Mouse anti-human β-actin antibody (1:5,000) was used as an internal standard. Blots were developed with horseradish peroxidase (HRP)-linked secondary antibodies (1:3,000) and visualized by the enhanced chemiluminescence (ECL) system using ImmunoStar Zeta (Wako, Osaka, Japan), and the bands were detected by LAS-4000 (GE Healthcare, Little Chalfont, UK). For the comparison of each protein expression, the bands were quantitatively analyzed by densitometry analysis using a software, Image J [39]. Each value relative to the one against β-actin band was depicted and standardized based on the results of controls cells (n=6). Then, the mean value in each condition was statistically compared. To know more about the subcellular localization and expression of PKM2 protein, HSC-4 cell fractionation was done according to the manufacture’s protocols using nuclear/cytosol fractionation kit (BioVision, Milpitas, CA, USA), then the cytoplasmic and nuclear fractions were applied for western blotting analyses as described above.

### RNA interference

PKM2 knockdown was carried out using small interfering RNA (siRNA) oligonucleotides (siPKM2) synthesized by Sigma-Aldrich (St. Loius, MO, USA). For negative control, siScramble (siScrbl) was also purchased from Sigma-Aldrich. siRNA was transfected into HSC-4 and SAS cells at 25 pmol/125 μl final concentrations with Screen Fect A plus (Wako, Osaka, Japan), using a forward transfection method according to the manufacture’s protocols.

### RT-qPCR analysis

RNA was prepared using ReliaPrep RNA Cell Miniprep System (Promega, Madison, WS, USA), and cDNA was prepared using the ReverTra Ace (Toyobo, Osaka, Japan), according to the manufacturer’s protocol. Real time quantitative PCR (RT-qPCR) was performed using Fast start essential DNA green master (Roche, Basel, Switzerland). The samples were analyzed, and message levels of *PKM2* or *TGIF2* were normalized to the corresponding *GAPDH* level. The PCR primer sequences used were as follows: *PKM2;* forward, 5’-TCCGCCGCCTGGCGCCCATTA-3’ and reverse, 5’-CTGACGAGCTGTCTGGGGAT-3’, *TGIF2;* forward, 5’-GTGCTGTTTCTGTCAAGCCA-3’ and reverse, 5’-AGCTCACCAGAACGCTATCA-3’, *GAPDH*; forward, 5’-ATCACCATCTTCCAGGAGCGAG-3’ and reverse, 5’-TGGCATGGACTGTGGTCATG-3’.

### Wound healing assay

Wounds were prepared by using Culture-Insert (2 well; ibidi, Madison, WS, USA). HSC-4 and SAS cells were cultured for 48 hours in each objective condition and removed the Culture-Insert. After 6 hours sustained culture, the area of remaining wounds was determined using Image J (National Institutes of Health, Bethesda, MD, USA). Then, the mean value of the closing ratio of wounded area (CR = (w-rw)/w x 100 (%), w: wounded area at the start point, rw: remaining wounded area) in each condition was statistically compared.

### Cell migration and invasion assay

HSC-4 and SAS cells cultured in serum free standard medium with or without siPKM2 in EMT (+) condition were seeded into each insert of the 24-well Falcon culture insert (#353097, Corning, NY, USA) for migration assay, or seeded into each insert of the 24-well Biocoat Matrigel Invasion Chamber (#351180, Corning) for invasion assay. FBS was added at the final concentration of 10% in each 24-well plate outer chamber to induce cell migration or invasion. After incubation for 72 hours, the cells remaining on the surface of the insert membranes were carefully removed with cotton swabs, and the cells that had migrated or invaded to the opposite sides of the membranes were stained with the Diff-Quik Kit (Sysmex Corp., Kobe, Japan). The total number of migrated or invaded cells obtained from 6 samples of each experiment were counted, and the median number was applied for statistical analyses.

### Statistical analysis

All data were expressed as the mean ± standard error of the mean (SEM). Student's t-test and Mann–Whitney U test were applied for the comparison between two groups. Kruskal-Wallis test and consecutive Mann–Whitney U test with a Bonferroni correction were applied for multiple comparisons. Statistical significance was set as ^*^p<0.05, ^**^p<0.01 and ^***^p<0.001.

## SUPPLEMENTARY MATERIALS FIGURES


