# Supplementary material for: Nuclear PKM2 promotes the progression of oral squamous cell carcinoma by inducing EMT and post-translationally repressing TGIF2
Source: Oncotarget. 2018 Sep 18;9(73):33745–61. doi: 10.18632/oncotarget.25850 (PMC6173467; doi:10.18632/oncotarget.25850)
Supplement: Supplementary file 1 [file oncotarget-09-33745-s001.pdf]

Nuclear PKM2 promotes the progression of oral squamous cell carcinoma by inducing EMT and post-translationally repressing TGIF2

SUPPLEMENTARY MATERIALS

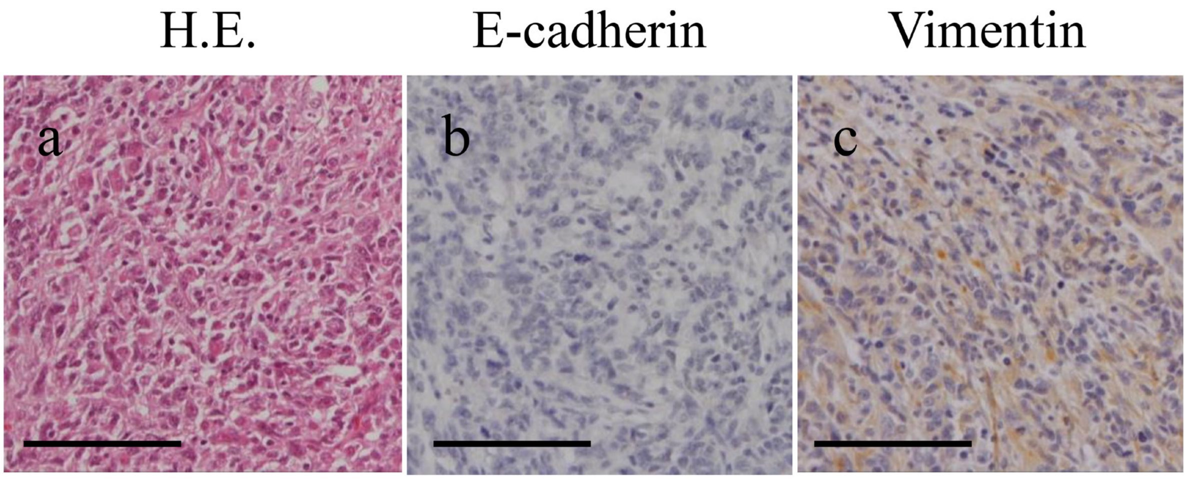

Supplementary Figure 1: EMT like changes in the OSCC specimen. Panels show H.E. staining **(a)**, and immunostaining of E-cadherin **(b)** and vimentin **(c)**, in spindle-shaped cancer cells. The repression of E-cadherin (b) and the expression of vimentin (c) are seen in spindle-shaped cancer cells. Scale bars: 100 µm.

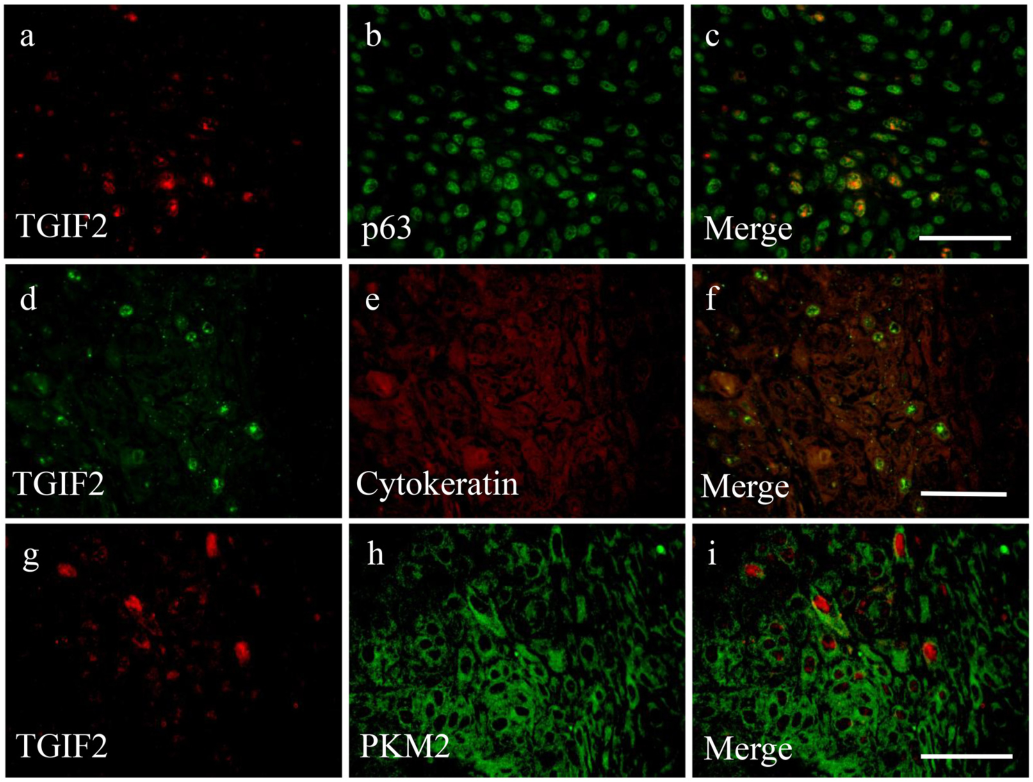

Supplementary Figure 2: Identification of PKM2 and TGIF2 expression in cancer cells in the same case shown in Figure 1A; g. Dual immunocytostaining of TGIF2 and basal cell marker p63 **(a, b, c)**, TGIF2 and epithelial marker cytokeratin **(d, e, f)**, and TGIF2 and PKM2 **(g, h, i)** is done. TGIF2 is expressed in the cancer cells that express basal cell marker p63 (a, b, c) and epithelial cell marker cytokeratin (d, e, f). Furthermore, TGIF2 is expressed in the cancer cells that express PKM2 (g, h, i). Scale bars: 100 µm.

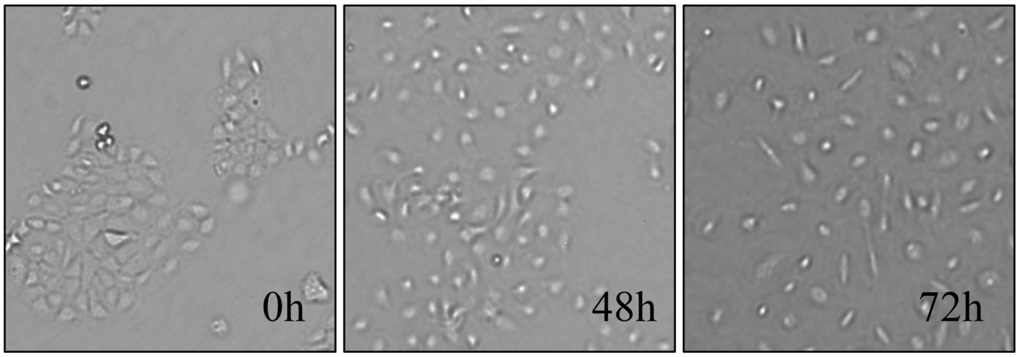

Supplementary Figure 3: Morphological changes of HSC-4 cells during EMT induction. HSC-4 cells show morphological changes from the round- or oval- to spindle-shaped feature in a time dependent manner (0 hour, 48 hours, 72 hours).

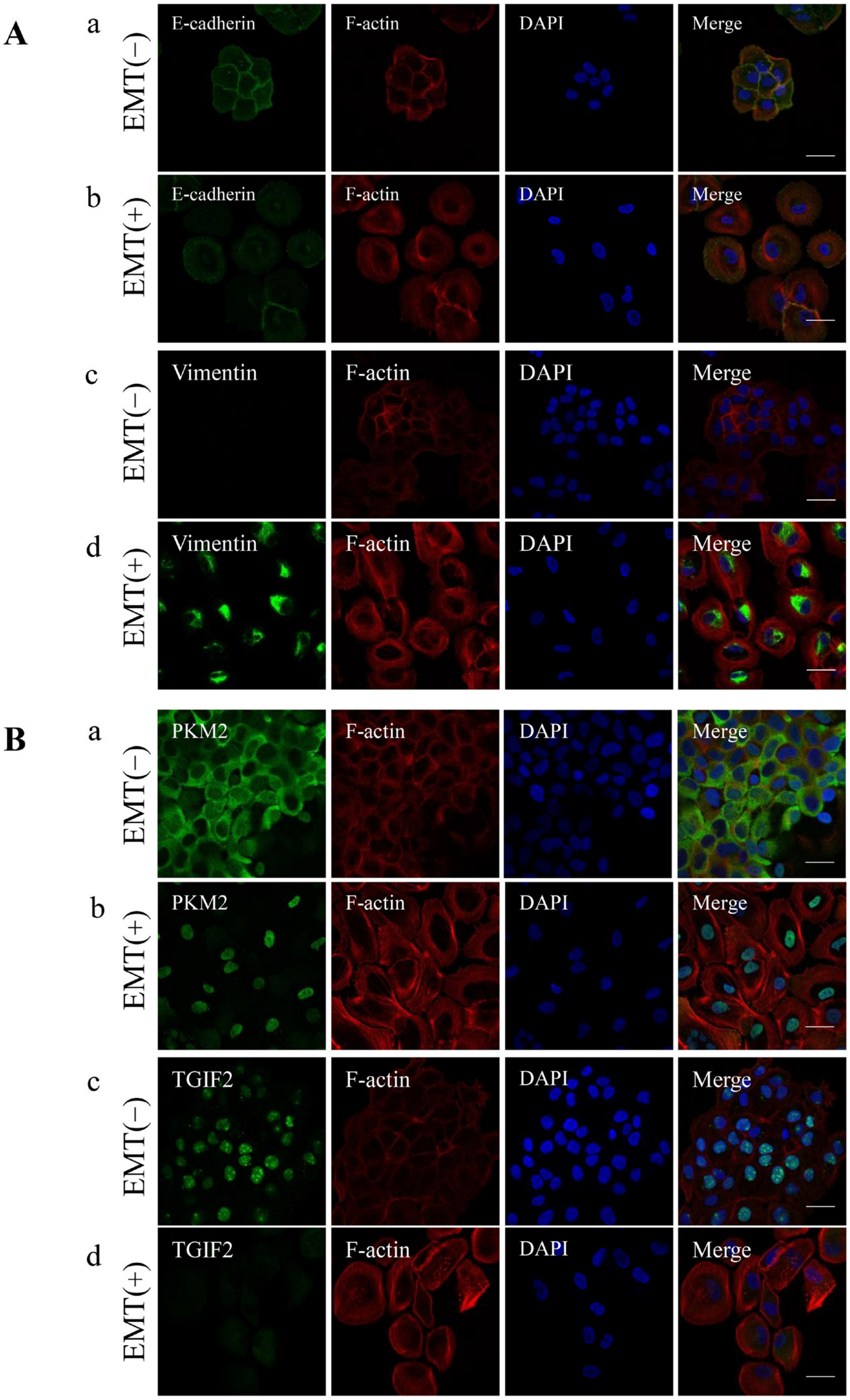

Supplementary Figure 4: EMT induction alters subcellular localization of PKM2 and expression of TGIF2, single-channel and merged images. (A) Immunofluorescent cytochemical staining of E-cadherin (a, b: green), vimentin (c, d: green), F-actin (a-d: red) and Nuclear DAPI (a-d: blue) for HSC-4 cells in EMT (-) (panels; a, c) and EMT (+) (panels; b, d). Single-channel and merged image panels are shown. Each merged image is shown in the right side in each column. **(B)** Immunofluorescent cytochemical staining of PKM2 (a, b: green), TGIF2 (c, d: green), F-actin (a-d: red) and Nuclear DAPI (a-d: blue) for HSC-4 cells in EMT (-) (panels; a, c) and EMT (+) (panels; b, d). Single-channel and merged image panels are shown. Each merged image is shown in the right side in each column. Scale bars in A, B: 20μm.

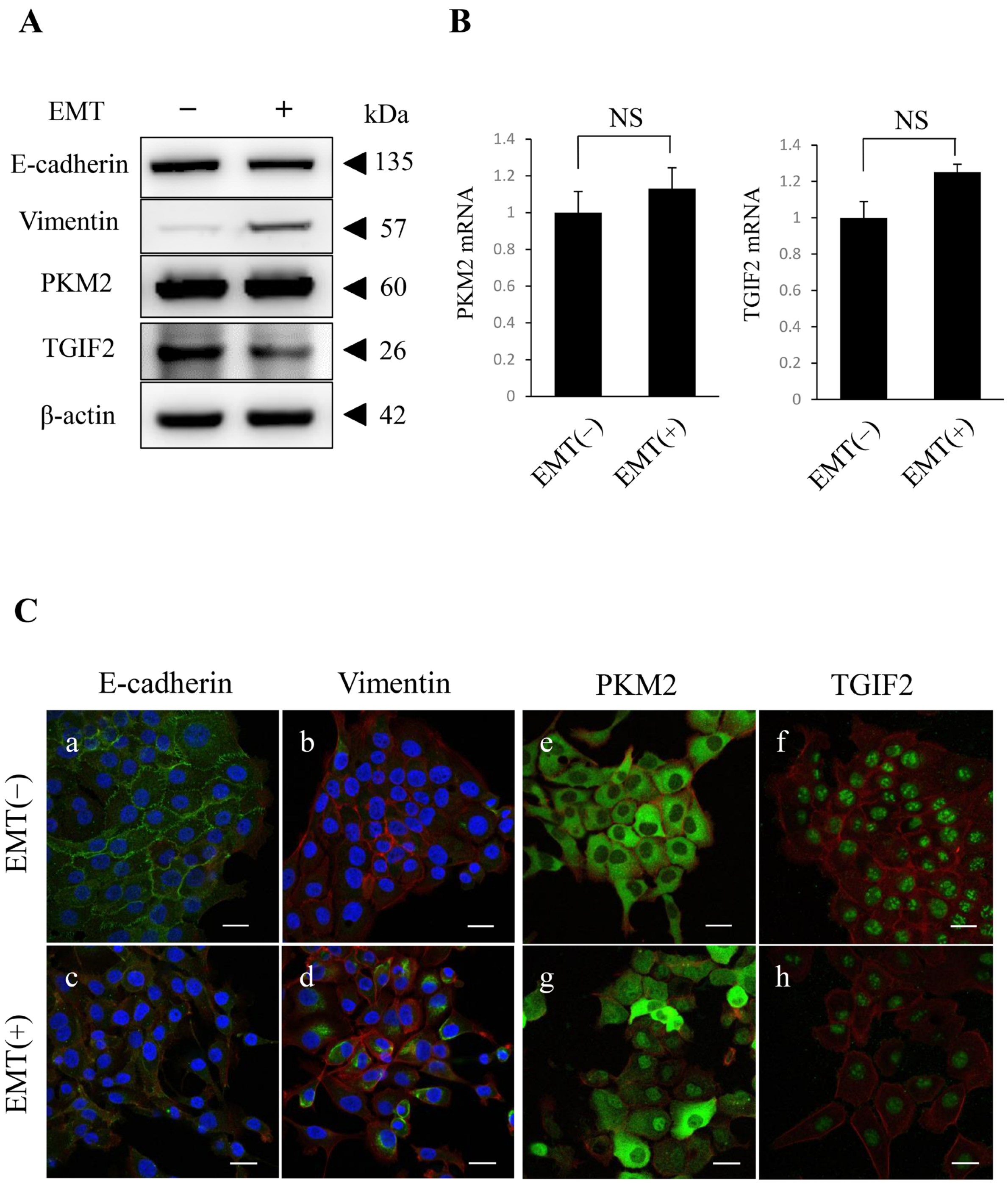

Supplementary Figure 5: PKM2 and TGIF2 expression in SAS cells in the EMT unstimulated condition (EMT (-)) and stimulated condition (EMT (+)). (A) Western blots of N-cadherin, E-cadherin, vimentin, PKM2 and TGIF2 in the EMT (-) and EMT (+). TGIF2 band in EMT (+) is weaker than that in EMT (-) although change of PKM2 bands is not apparent in between EMT (-) and (+). Molecular weight is pointed by an arrowhead. **(B)** RT-qPCR analyses for PKM2 and TGIF2 mRNA expression in EMT (-) and (+). No statistical difference is seen in both PKM2 and TGIF2 mRNA expression in between EMT (-) and (+). These results reveal mismatch in between TGIF2 protein and mRNA expression. **(C)** EMT induction is confirmed by repression of E-cadherin expression (c) and induction of vimentin (d) in EMT (+). EMT induction alters subcellular localization of PKM2 from cytoplasm (e) to nucleus (g) and nuclear expression of TGIF2 to be repressed (h). Scale bars: 20 μm.

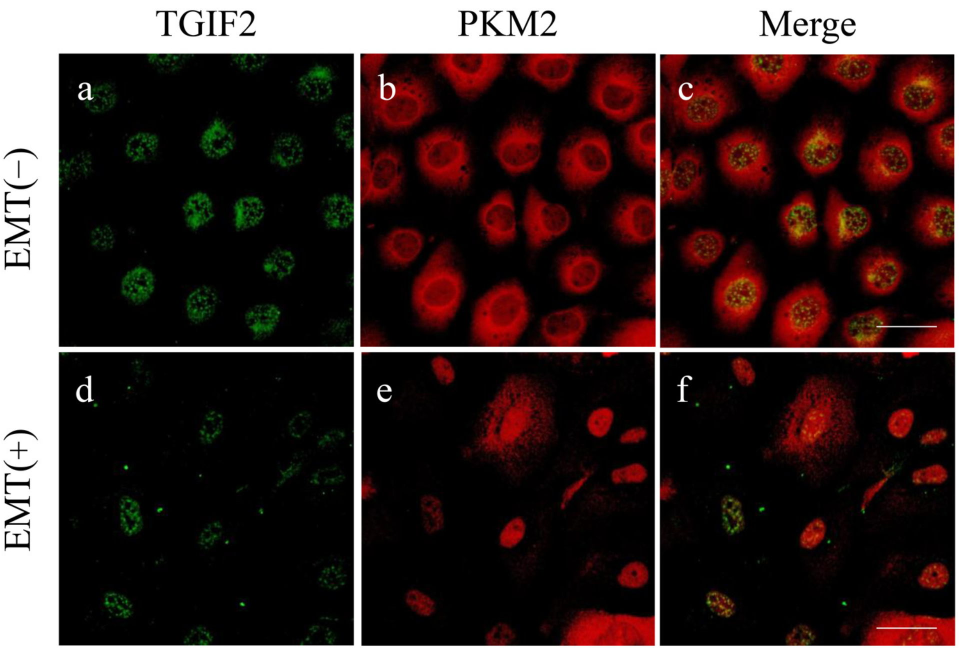

Supplementary Figure 6: Immunofluorescent cytochemical analyses of relative expression of TGIF2 and PKM2 by dual immunostaining. The repression of nuclear TGIF2 expression (d) in the nucleus showing localized expression of PKM2 (e) in EMT induced HSC-4 cells is confirmed by the TGIF2 and PKM2 dual immunocytochemistry. Scale bars: 20 µm.

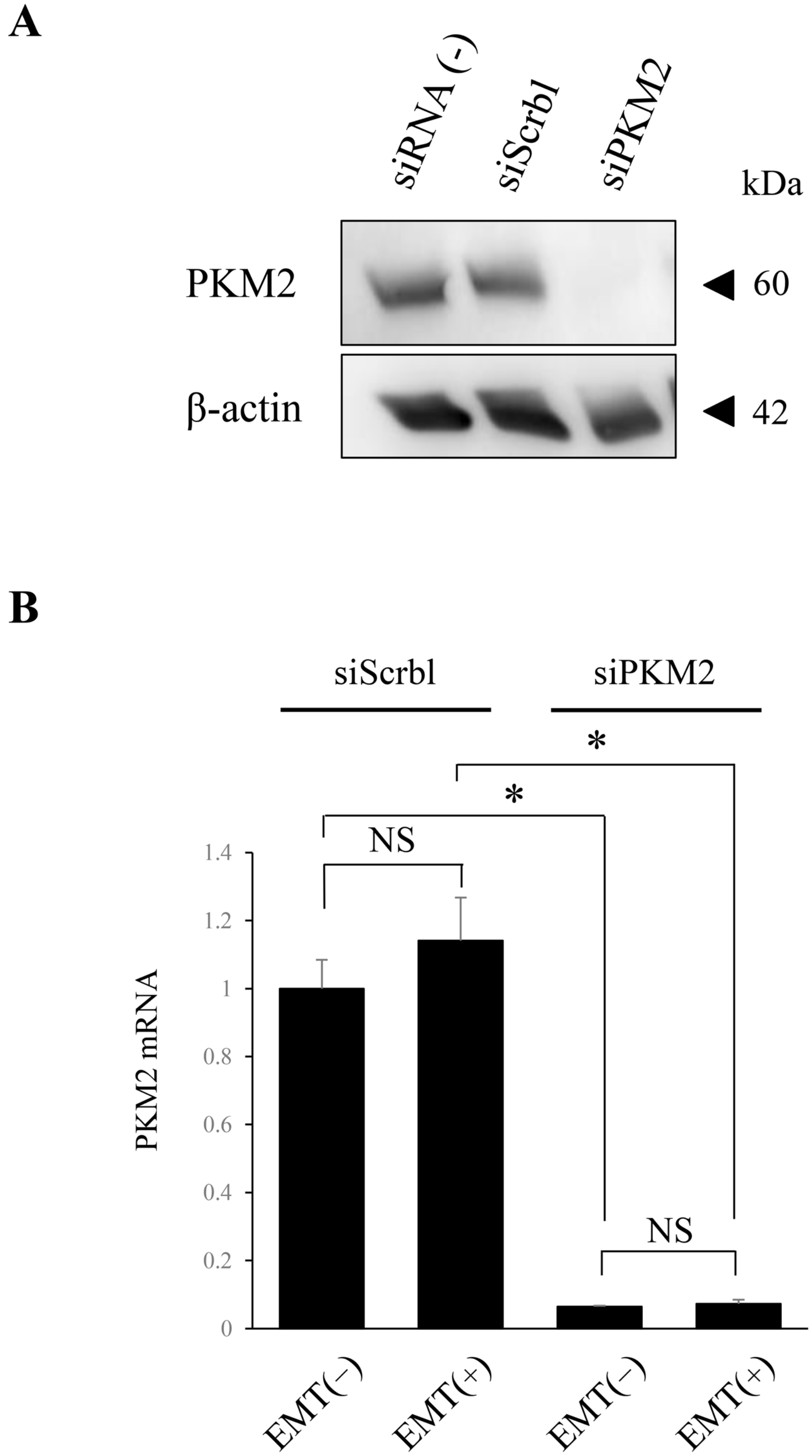

Supplementary Figure 7: PKM2 knockdown in HSC-4 cells. **(A)** In western blotting analyses, PKM2 expression is distinctively repressed in PKM2 knocked down cells (siPKM2). Molecular weight is pointed by an arrowhead. **(B)** Real-time qPCR analyses show that the expression of PKM2 mRNA in HSC-4 cells with siScrbl transfection is significantly repressed in HSC-4 cells with siPKM2 transfection in both EMT (-) and EMT (+) conditions (n=4). Statistical significance was set as $^{*}p<0.05$.

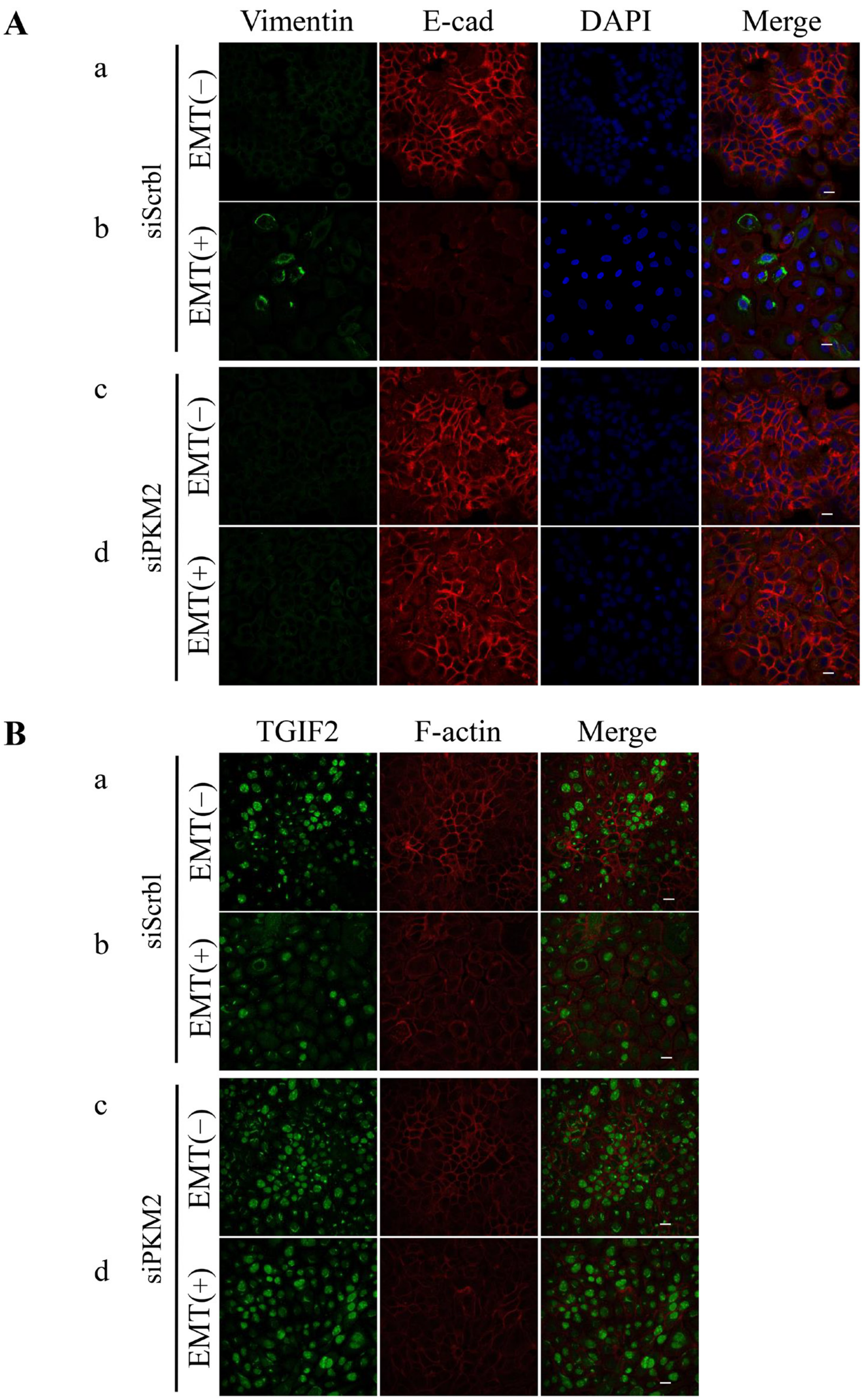

Supplementary Figure 8: Inhibition of EMT induction by siPKM2 in EMT stimulated HSC-4 cells, single-channel and merged images. **(A)** Immunofluorescent cytochemical staining of vimentin (green), E-cadherin (E-cad; red) and Nuclear DAPI (blue) for HSC-4 cells in EMT(-) (a, c) or EMT(+) (b, d) with siScrbl (a, b) or siPKM2 (c, d) transfection. Single-channel and merged image panels are shown. Each merged image is shown in the right side in each column. **(B)** Immunofluorescent cytochemical staining of TGIF2 (green) and F-actin (red) for HSC-4 cells in EMT (-) (a, c) or EMT (+) (b, d) with siScrbl (a, b) or siPKM2 (c, d) transfection. Single-channel and merged image panels are shown. Each merged image is shown in the right side in each column. Scale bars in A, B: 20μm.

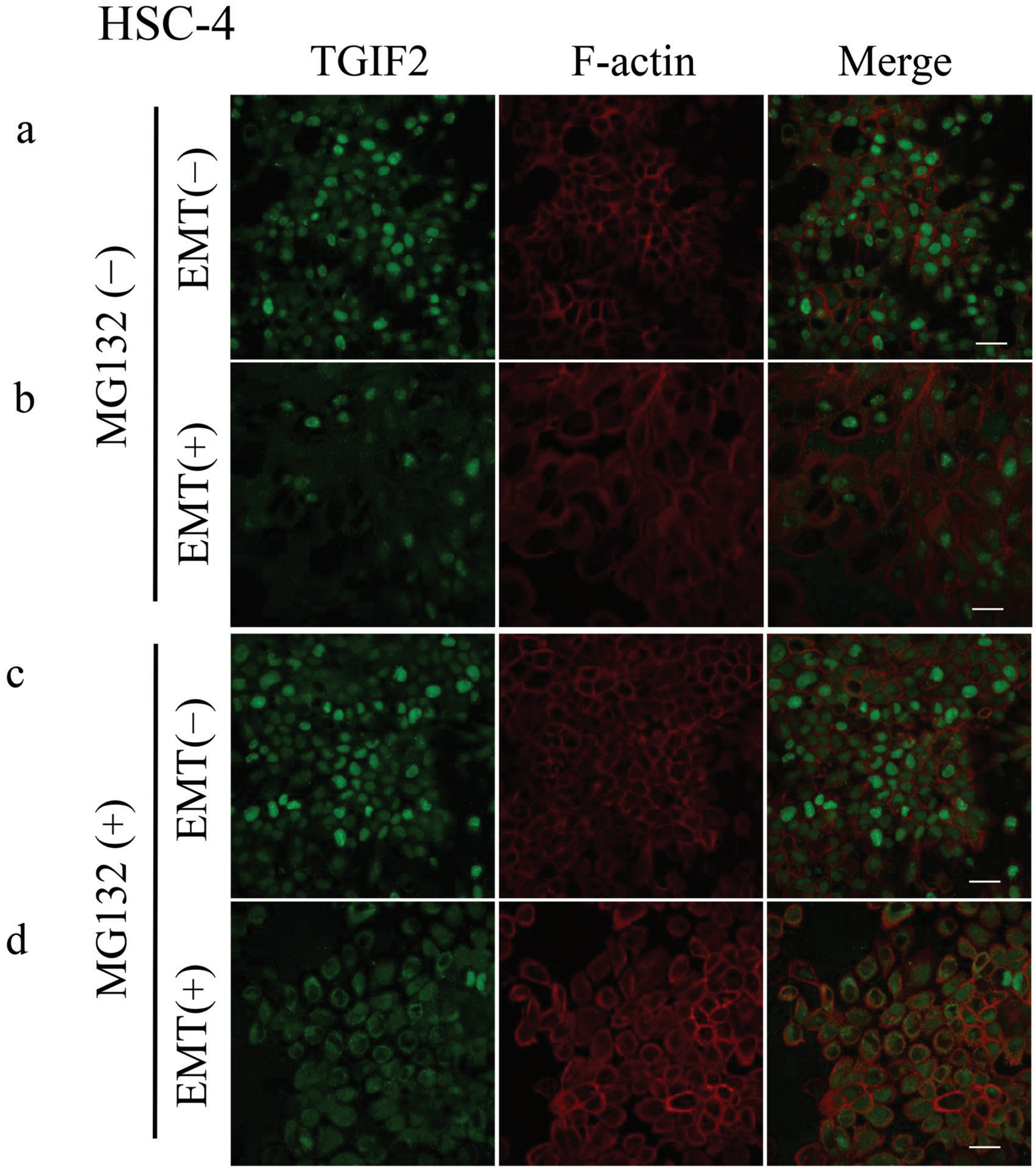

Supplementary Figure 9: Analyses of the TGIF2 expression and subcellular localization in HSC-4 cells in the EMT stimulated and a proteasome inhibitor MG132 treated condition, single-channel and merged images. Immunofluorescent cytochemical staining of TGIF2 (green) and F-actin (red) for HSC-4 cells in EMT (-) **(a, c)** or EMT (+) **(b, d)** with MG132 untreated (MG132 (-)) (a, b) or treated (MG132 (+)) (c, d) condition. Single-channel and merged image panels are shown. Each merged image is shown in the right side in each column. Scale bars: 20μm.

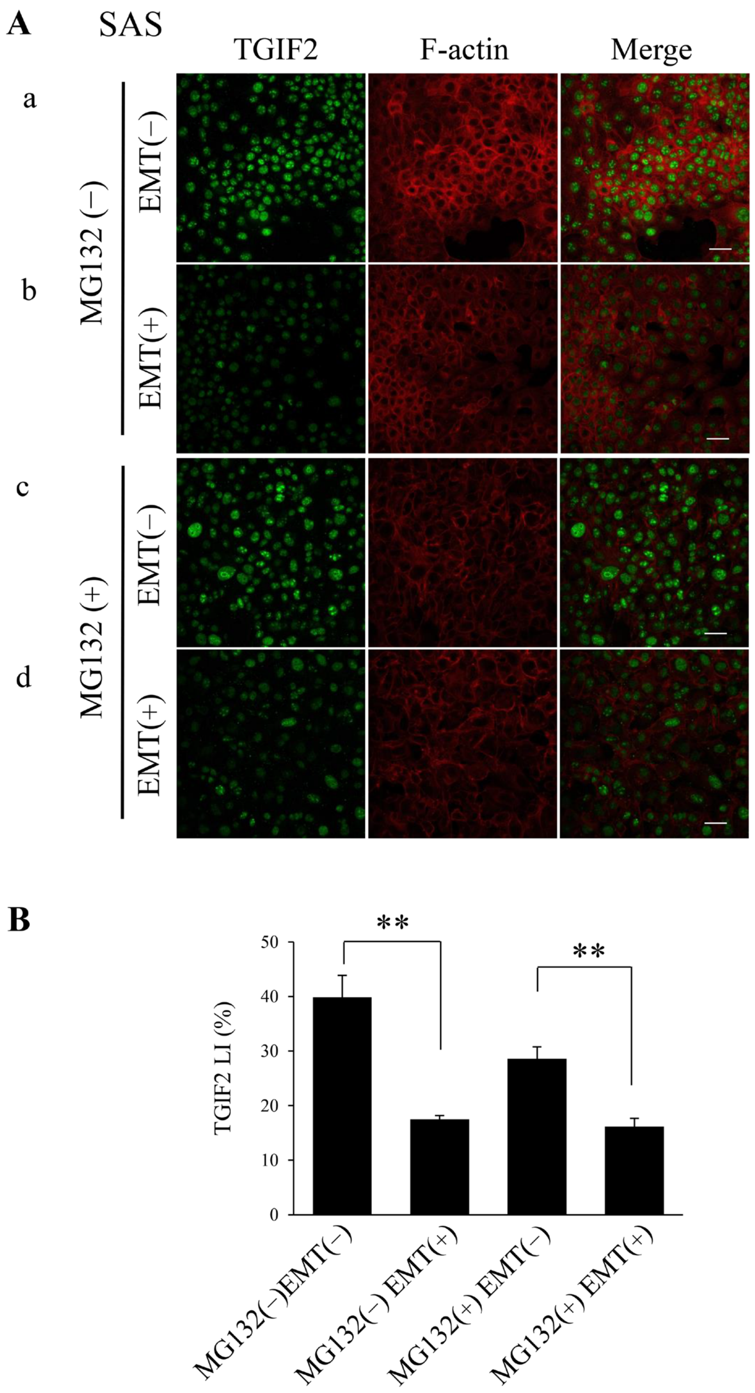

Supplementary Figure 10: Analyses of the TGIF2 expression and subcellular localization in SAS cells in the EMT stimulated and a proteasome inhibitor MG132 treated condition, single-channel and merged images. (A) Immunofluorescent cytochemical staining of TGIF2 (green) and F-actin (red) for SAS cells in EMT (-) (a, c) or EMT (+) (b, d) with MG132 untreated (MG132 (-)) (a, b) or treated (MG132 (+)) (c, d) condition. Single-channel and merged image panels are shown. Each merged image is shown in the right side in each column. Scale bars: 20μm. **(B)** Analyses of MG132 effect on nuclear TGIF2 expression in SAS cells in EMT (-) and EMT (+). Significant decrease of TGIF2 LI is seen in MG132(-)EMT(+) and MG132(+)EMT(+) compared to those in MG132(-)EMT(-) and MG132(+)EMT(-), respectively. From these findings, nuclear TGIF2 expression is significantly repressed in EMT (+) regardless of MG132 treatment. Statistical significance was set as $^{**}p<0.01$.

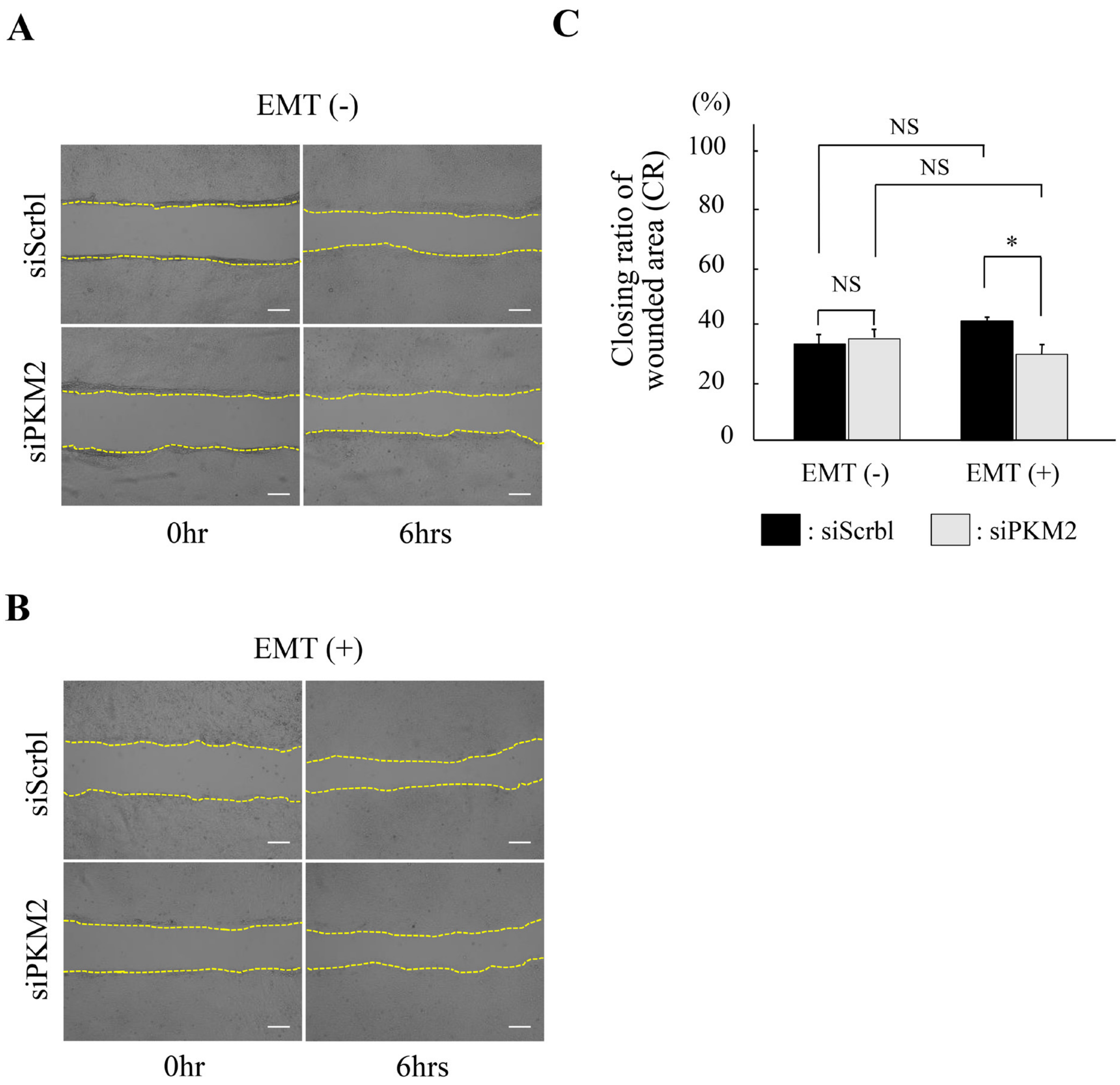

Supplementary Figure 11: Functional analyses of PKM2 in SAS cells. Wound healing assay to evaluate the ability of SAS cells migration in siScramble (siScrbl) or siPKM2 transfected cells. The closing pattern of wounded area is displayed in the panels of each EMT unstimulated (EMT (-)) **(A)** or stimulated (EMT (+)) **(B)** condition. Upper panels show siScrbl transfected cells and lower panels show siPKM2 transfected cells in EMT (-) (A) or EMT (+) (B). The culture is stopped after 6 hrs (each right column) from the start 0 hr (each left column) (A, B). Bars indicate the closing ratio of wounded area (CR) in siScrbl (black bars) or siPKM2 (gray bars) transfected cells in EMT (-) or EMT (+) (n=6) **(C)**. Statistical significance was set as $^{*}p<0.05$.
